# Time-window into the transcrustal plumbing system dynamics of Dominica (Lesser Antilles)

**DOI:** 10.1038/s41598-021-90831-1

**Published:** 2021-06-01

**Authors:** Lea Ostorero, Georges Boudon, Hélène Balcone-Boissard, Daniel J. Morgan, Thiebaut d’Augustin, Clara Solaro

**Affiliations:** 1 Université de Paris, Institut de physique du globe de Paris, CNRS, F-75005 Paris, France; 2 Institut des Sciences de la Terre de Paris (ISTeP), UMR 7193, CNRS-Sorbonne Université, Paris, France; 3 Institute of Geophysics and Tectonics, School of Earth & Environment, University of Leeds, Leeds, LS2 9JT UK

**Keywords:** Natural hazards, Solid Earth sciences

## Abstract

Dominica, one of the most magmatically active islands of the Lesser Antilles through its four active volcanoes, is likely host under its central part, below Morne Trois Pitons–Micotrin, to a well-established transcrustal mush system. Pre-eruptive spatiotemporal magma dynamics are examined for five, explosive, pumiceous eruptions of this volcano in the last 24 kyrs through a combined Crystal System Analysis and intracrystalline Fe–Mg interdiffusion timescales modelling approaches. Before all eruptions, two magmatic environments of close compositions have interacted. These interactions began ~ 10–30 years prior to the four smaller of these eruptions, with more sustained mixing in the last decade, accelerated in the last 2 years. This contrasts with the largest pumiceous eruption, involving deeper magmas, with magma interaction starting over roughly a century but with various patterns. This suggests a possibility that increasing reactivation signals could be registered at the surface some years before future eruptions, having significant implications for volcanic risk mitigation.

## Introduction

Volcanic eruptions are among the most energetic events on Earth. In a subduction context, eruptions are generally explosive and involve differentiated magmas. These magmas can display variable eruptive styles depending on the volume of magma involved and the relative behaviour of the melt and volatile components during ascent. Deciphering the dynamics of magmas in the crust contributes to a better understanding of magma plumbing system architecture and behaviour, which leads in turn to better management of volcanic crises prior to an eruption^1,2^. Over the last 10 years, physical models suggest that magma chambers are not easy to form and are unstable, contrary to earlier views of these storage areas, with a stable magma chamber dominated by the presence of liquid^3^. Recent works have shown that magmatic reservoirs are likely to be heterogeneously distributed in space and time through the whole crust, as multiple lenses of liquid-dominated magmas, possibly interconnected, surrounded by a mush that is mainly composed of crystals (> 50%) and plutonic rocks derived from the same magmatic reservoirs^4–6^.

Spatiotemporal magma storage and dynamics can be investigated by coupling timescale modelling^7^ and the study of the diversity of crystal compositions (in particular zonations), following the Crystal System Analysis approach (CSA)^8^. A CSA approach aims to decipher the record of different pathways experienced by crystals, considering them as sequential of changes in magmatic environments (ME), each defined by constant set of intensive thermodynamic variables such as pressure (P), temperature (T), composition and volatile species fugacities^7,9–12^. It does this by using connectivity diagrams to create a framework in which to organize and interpret the compositional information from crystal cores to the rims^7,9–11^.

Such investigations have successfully been applied on Etna, using olivine crystals to identify magma ponding zones and their varying connections in space and time prior to eruption^7,9–11^. In parallel, following pioneering works on modelling intracrystalline diffusion^13–17^ to estimate timescales for changes in magma storage conditions, many applications for diffusion chronometry methods have been found^15,18–29^. Studies have focused on systems including ridge volcanism^13,30^ and arc magmatism^16,24,25,31,32^.

Readjustments in mineral-melt equilibria during crystallization can cause a variety of zonation patterns to be formed in growing crystals. These readjustments arise following changes in one or more of the key intensive variables — P, T, oxygen fugacity (*f*O_2_), melt composition or volatile abundance^33^. Following the creation of compositional gradients in crystals, intracrystalline diffusion will act to homogenize and re-equilibrate a zoned crystal. Ionic diffusivity in silicates depends on various parameters such as the chemical element, the specific mineral, the crystallographic direction, and the ambient T, P, and *f*O_2_^21,29^. Diffusion is a thermally-activated process, so the abrupt temperature drop during the eruption leads to effectively instantaneous, cessation of diffusive re-equilibration processes and a “fossilization” of the information linked to pre-eruptive processes in magmatic reservoir. Diffusion chronometry assumes that the diffusion of the elements studied in the mineral must be fast enough to partially erase the zonation but slow enough so that the crystal does not totally equilibrate under the new conditions, allowing temporal information to be recovered^17^.

**Figure 1:**
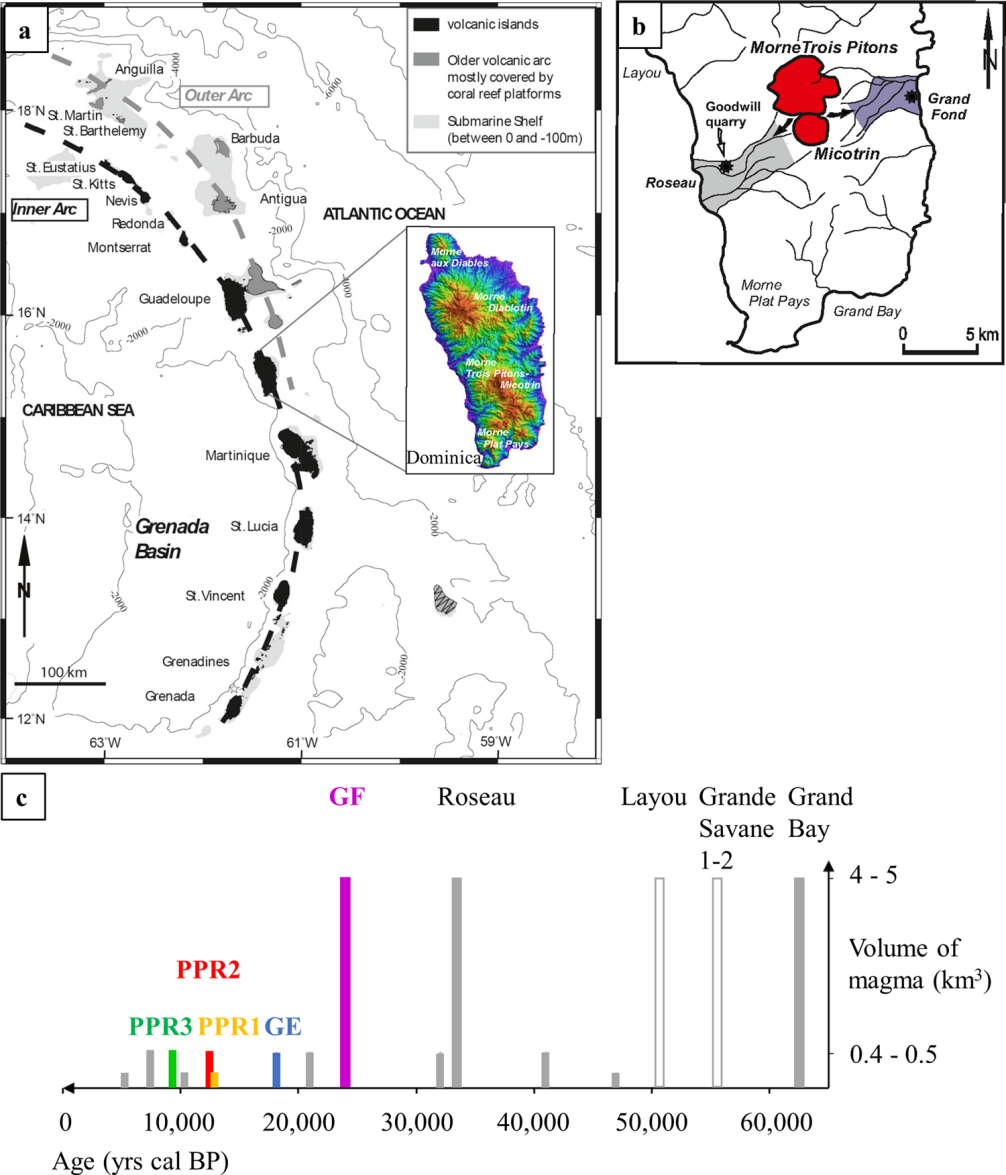
Dominica eruptions. (**a**) The Lesser Antilles arc^60–62^ results from the subduction of the northern and southern Atlantic plates beneath the Caribbean plate at a rate of ~ 2 cm/yr^63^. Black: volcanic islands, grey: coral platforms; the map has been modified from a previous study^39^. Bathymetry is in light grey^60^. Inset: Shuttle Radar Topography Mission (SRTM) topography of Dominica with four actives volcanic centers (Morne aux Diables, Morne Diablotins, Morne Trois Pitons-Micotrin and Morne Plat Pays, from north to south) and the largest magmatic production of the Lesser Antilles arc (~ 40 km^3^ for the last 100 kyrs) (Courtesy of Dr. Ian C.F. Stewart). (**b**) Map of southern Dominica and location of the studied deposits. From the Morne Trois Pitons-Micotrin volcano, in purple: Grand Fond (GF) large explosive eruption deposits in the valley, outcrop at (15°21′57″N, 61°15′16″W, marked by a star on the map) and Goodwill Entrance (GE), PPR1, 2 and 3, in the Roseau Valley (Goodwill quarry: 15°18′32″N, 61°23′03″W, star on the map), in grey: Roseau ignimbritic deposits^34^. The maps of (**a**,**b**) have been done with CorelDraw Graphics Suite 2020 (https://www.coreldraw.com/fr/). (**c**) Chronology of the pumiceous eruptions from Morne Trois Pitons-Micotrin for 70 kyrs. Rectangles: filled ones: eruptive deposits from Morne Trois Pitons–Micotrin and empty ones: eruptive deposits from Morne Diablotins, their height depends on the magma volume (km^3^)^34^. Colors: purple for Grand Fond, blue for Goodwill, yellow for PPR1, red for PPR2, green for PPR3 and grey for the unstudied eruptions. The large explosive eruptions involve 4–5 km^3^ of magma Dense Rock Equivalent (DRE); the voluminous pyroclastic density currents, which are several tens of meters thick in the main valleys, are more or less welded. The small explosive eruptions involve magma volumes of at least one order of magnitude lower than the large explosive ones^34^.

Our study focuses on Dominica (Lesser Antilles arc), which is the sole island of the arc exhibiting several active volcanic centers and a high magma production rate (Fig. 1a,b). Volcanic activity involving evolved (andesitic-dacitic) magmas is associated with two volcanoes in the central part of Dominica. On Morne Trois Pitons-Micotrin volcanic center, two large pumiceous eruptions (Volcanic Explosivity Index (VEI) of 5^34,35^) occurred 33 and 24 kyrs ago^34,36,37^. They each generated a fallout layer from a Plinian phase followed by voluminous overflowing pyroclastic density currents sometimes forming welded ignimbritic deposits found in multiple valleys^34^. In the last 20 kyrs, several pumiceous eruptions of smaller magnitude (VEI of 4^34,35^) have occurred, also originating from Morne Trois Pitons-Micotrin volcanic center. They each generated a Plinian phase with a similar deposit sequence, with dominant pumice fallout followed by low volume of pyroclastic density currents due to collapse of the eruptive column^34^.

In this study, the aim is to examine the spatiotemporal dynamics of the magmas in the plumbing system that fed the successive pumiceous eruptions (one large and four small ones) of the last 24 kyrs of Morne Trois Pitons-Micotrin. These eruptions were chosen because of their recent ages, a good chronostratigraphic control, well-preserved deposits and homogeneous compositions. This permits an investigation of how the storage system of this volcano changes in time and its dynamics, following a larger explosive eruption associated with deeper magma supply (12–16 km^38^) and smaller eruptions of magmas stored more shallowly (2–8 km^38,39^). Here, a detailed investigation of orthopyroxene compositions across the last five pumiceous eruptions allows identification of the magma transfer pathways and the existence of disrupting events related to magma remobilisation at depth, by adapting the CSA approach as used for previous large explosive eruptions of Dominica^12^. Timescales associated with the pre-eruptive dynamics were obtained by modelling Fe–Mg interdiffusion between cores and rims^12,25^. Orthopyroxenes of these eruptions are key witnesses of magmatic processes at depth, and are well-suited for study because they are the best-preserved crystals, with recorded crystal compositions sensitive to temperature variations as determined via experimental works^38,40^. Results are then compared to those of other large explosive eruptions of Dominica, permitting a broader understanding of the transcrustal magmatic system both at this highly active volcanic island and more generally with respect to subduction zone volcanism, which may provide insights useful for volcanic hazard mitigation in Dominica and more generally worldwide.

## Previous works

Field study of Dominica eruption products shows that the magma volumes emitted are in the order of 4–5 km^3^ Dense Rock Equivalent (DRE) for larger pumiceous eruptions and roughly one order of magnitude lower (< 1 km^3^ DRE) for the smaller eruptions^34^.

Furthermore, the storage system beneath Morne Trois Pitons-Micotrin includes two main storage depths^12,38,39^: at ~ 2–8 km depth (50–200 MPa), based on H_2_O-CO_2_ concentrations measured in melt inclusions^39^ for the recent small explosive eruptions reservoirs and ~ 12–16 km (300–400 MPa) based on experimental petrology^38^ and melt inclusions data^38^ for the voluminous pumiceous eruptions reservoirs (Supplementary notes on previous results are available in Supplementary material).

Whole rock compositions of all the studied magmas are andesitic-dacitic, whereas glass compositions are rhyolitic^34,38,39,41^. The pumices possess similar phenocryst abundances of ~ 30 vol% with a dominant proportion of plagioclase (~ 21 vol% for the large eruptions) followed by orthopyroxenes (~ 5% for the large eruptions) and in smaller proportions, clinopyroxenes, Fe-Ti oxides and amphiboles in some units^38,39^.

## Results

### Orthopyroxene zonation: type and proportions

We focus our study on five eruptions from Morne Trois Pitons-Micotrin^34^: the last voluminous pumiceous eruption of Grand Fond (24 046 ± 357 yr cal BP) and four of the smaller explosive eruptions that occurred in the last 20 kyrs: Goodwill Entrance (Goodwill): 18 095 ± 205 yr cal BP, “Plinian Post Roseau” 1 (PPR1): 12 619 ± 88 yr cal BP, PPR2: 12 513 ± 129 yr cal BP and PPR3: 9 391 ± 94 yr cal BP (Fig. 1a–c and Supplementary Figure S1).

**Figure 2:**
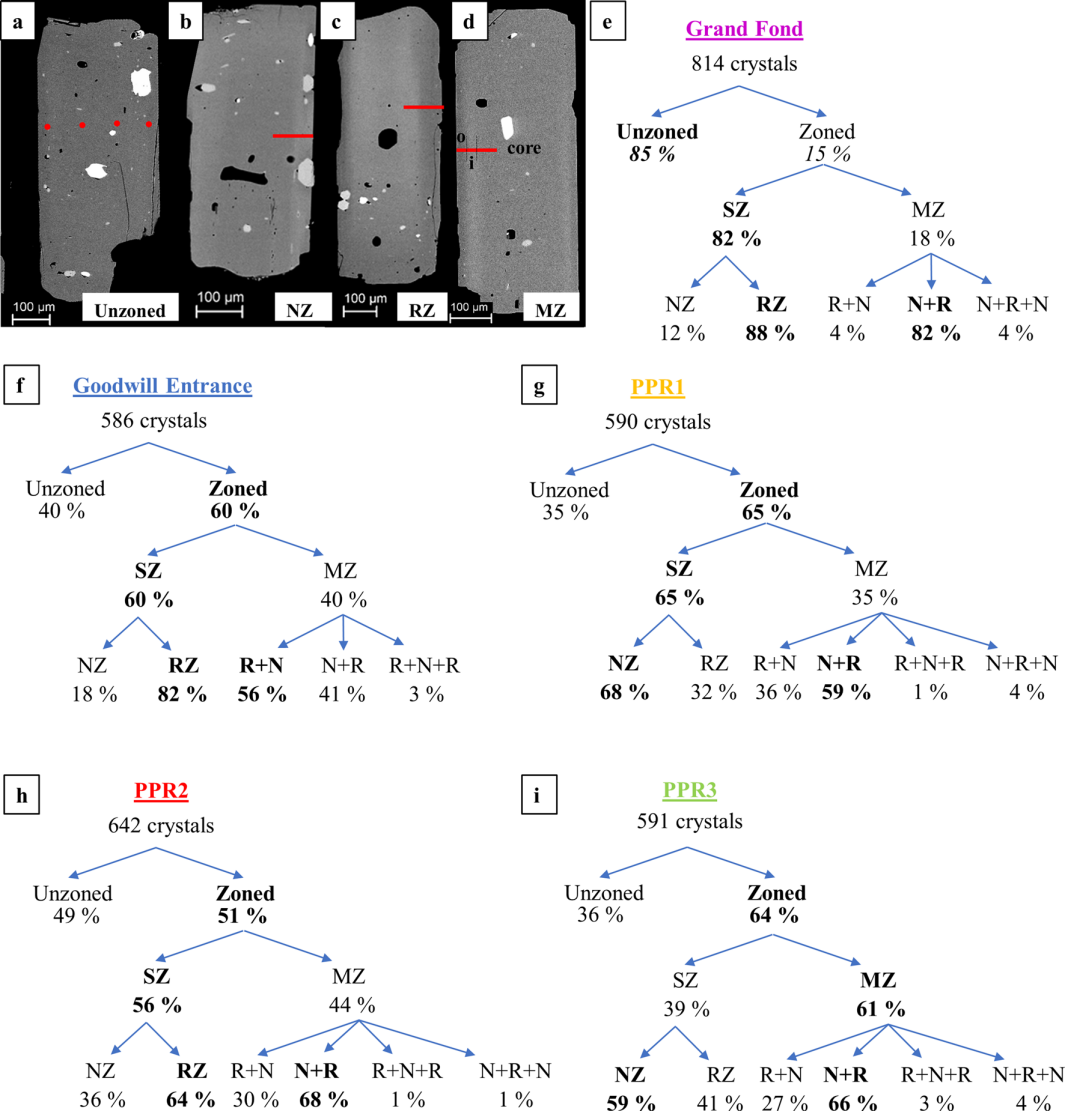
The different zonations identified in the opx cargo: images and proportions. SEM images (Sorbonne Université, Paris). (**a**) Unzoned crystal. Two types of single-zoned opx (SZ) have been identified: normal-zoned (NZ, **b**), with a Mg-rich core (darker zone on a grayscale image) and Fe-rich rims (clearer in grayscale), or reverse-zoned (RZ, **c**), with inversely a Fe-rich core and Mg-rich rims. (**d**) Multiple-zoned opx (MZ), either reverse + normal (R + N) or normal + reverse (N + R) or with a third rim (N + R + N or R + N + R, Supplementary Figure S3). The red line shows the location of the electron microprobe profiles (EPMA) and the core, inner (“i”) and outer rim (“o”) zones are annoted on the multiple-zoned opx (**d**). From (**e**) to (**i**) (drawn with Microsoft Office suite 2019 Version 1808; https://www.microsoft.com/fr-fr/microsoft-365): proportions of the zonations identified in the opx of Grand Fond in the 355–500 µm fraction (**e**) and in the opx of the small explosive eruptions of the three fractions studied all together (355–500, 250–355 and 125–250 µm fraction, **f** to **i**). 4% of the multiple zoned crystals studied had a third rim (less than 5 crystals per eruption). However, this third rim, though existing in the crystal, does not form a plateau in the compositional profiles (< 10 µm), so they will not be considered in the following results (Supplementary Figure S3). These last rims could correspond to very shorts events prior to the eruptions.

A total of 3224 orthopyroxene (opx) crystals were studied for the five eruptions. Zoned crystals of each eruption were classified according to the type of zonation observed, single zonation (either normal or reverse zonation) and multiple zonation (i.e. Oscillatory zonation) (Fig. 2a–d, Supplementary Table S1). In Grand Fond, of 814 opx studied, only 15% are zoned, with a prevalence in single-zoned and particularly in reverse-zoned crystals compared to normal-zoned ones (Fig. 2e). In the four small explosive eruptions, of the 2410 opx studied, the proportions of zoned crystals are considerably higher (between 51 and 65% of the population being zoned) (Fig. 2f–i). These proportions are considered to be representative and unbiased as the opx were handpicked and mounted randomly before determination of zoning by Scanning Electron Microscopy (see “Material and methods”). In more detail, zoning patterns are more complex for the small explosive eruptions compared to Grand Fond with a higher proportion of multiple-zoned crystals varying between 35 and 61% of opx crystals examined. In the zoned crystals, the proportions of single-zoned vary from 39 to 65% (Fig. 2f–i) with a proportion of normal-zoned from 18 to 68% and reverse-zoned from 32 to 82% (Fig. 2f–i). The multiple-zoned crystals show a dominance of normal zonations followed by reverse zonations (from core to rim) with the exception of Goodwill that shows a higher proportion of reverse then normal zonations (Fig. 2f–i).

### Orthopyroxene zonation: compositions

For Grand Fond, 49 core to rim compositional profiles were measured using an electron microprobe micro-analyzer (EPMA): 29 in unzoned crystals and 20 in zoned crystals (single- or multiple-zoned). For the four smaller eruptions, 258 profiles have been performed (72 for Goodwill, 59 for PPR1, 61 for PPR2 and 66 for PPR3). All profiles in zoned opx were acquired perpendicular to the zone boundaries and to the long axes of the crystals, far from the corners to reduce the impact of 3-D diffusion effects or recrystallization along the c-axis (Fig. 2b–d)^25,42,43^.

**Figure 3:**
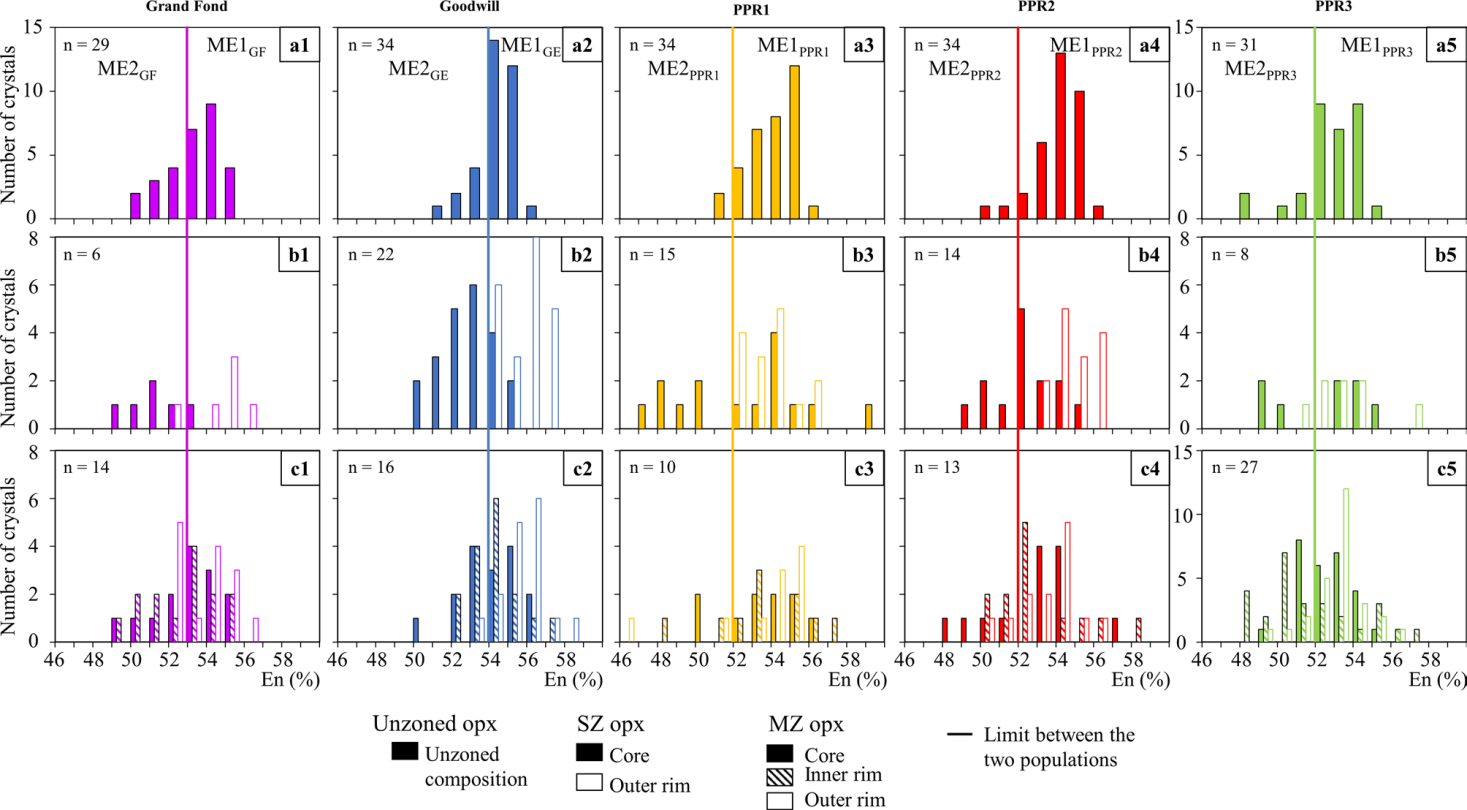
Frequency histograms of the En content of unzoned (**a1**–**a5**) and zoned opx of the five studied eruptions (single-zoned (SZ) opx: **b1**–**b5** and multiple-zoned (MZ) ones: **c1**–**c5**). These frequency histograms highlight two populations of opx: a first population of zoned opx with cores that have the same composition as the majority of the unzoned opx (population 1 in Fig. 4) and zoned opx with cores with lower En contents (less magnesian) than the unzoned opx (population 2 in Fig. 4). The boundary between these two populations are shown with a line on the Figure: En_53_ for Grand Fond, En_54_ for Goodwill and En_52_ for PPR1 to 3. The color for each eruption is the same as in Fig. 2. The magmatic environments (ME) discussed afterwards are specified here for each eruption. n: number of opx analysed.

**Figure 4:**
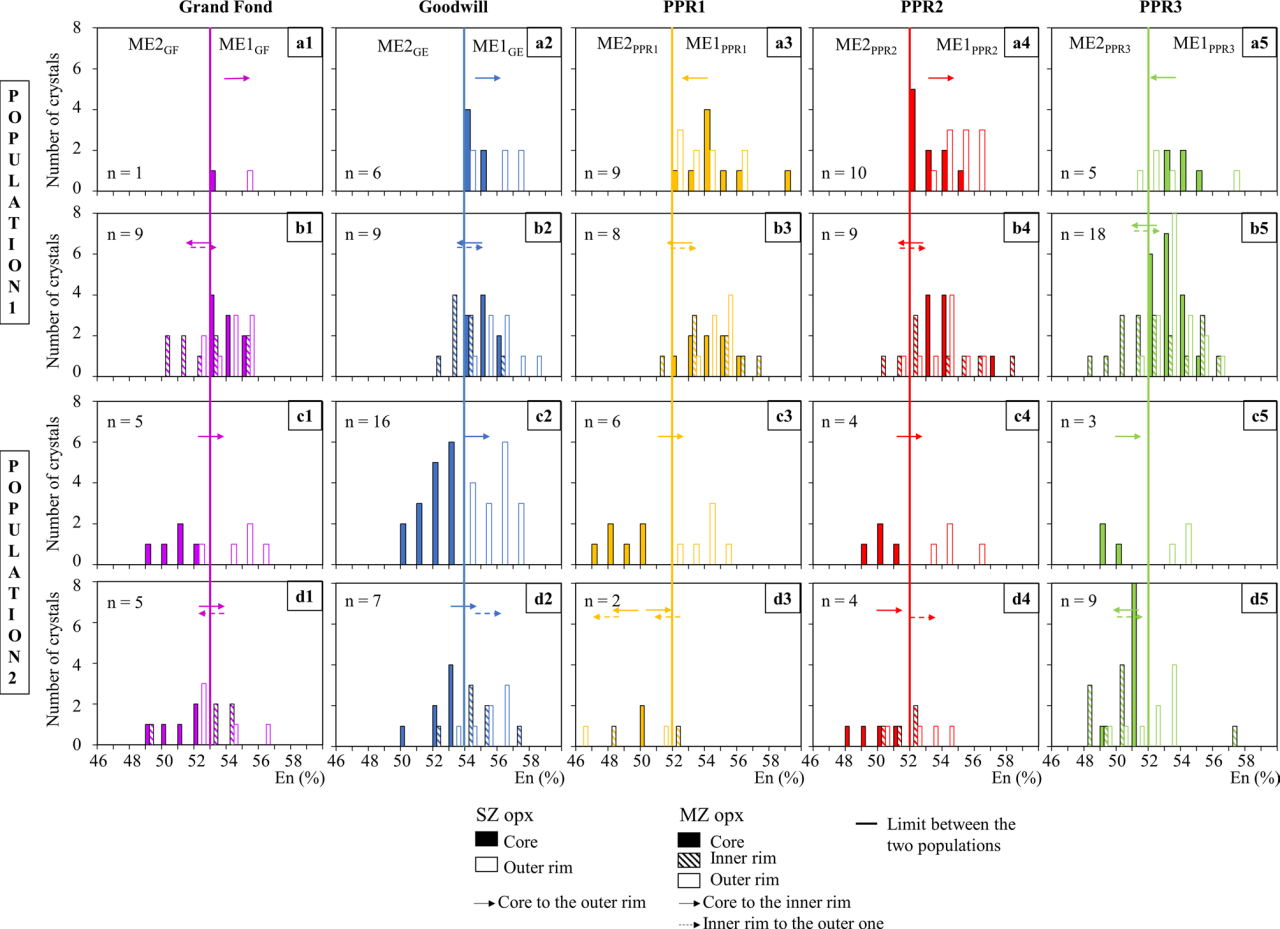
Frequency histograms of the En content of zoned opx of the five studied eruptions. The two populations of opx identified are the same as in Fig. 3. Population 1: cores ≥ En_53_ for Grand Fond, En_54_ for Goodwill and En_52_ for PPR1, 2 and 3 and population 2: cores < En_53_ for Grand Fond, En_54_ for Goodwill and En_52_ for PPR1, 2 and 3. The color for each eruption is the same as in Fig. 3. (**a1**) to (**a5**) single-zoned (SZ) opx of population 1; (**b1**) to (**b5**) multiple-zoned (MZ) opx of population 1; (**c1**) to (**c5**) SZ of population 2; (**d1**) to (**d5**) MZ of population 2. The arrows underline the major path from the core to the rim(s) recorded by the SZ opx or, for MZ, from the core to the inner rim and dashed line: inner rim to the outer one of the MZ opx. The magmatic environments (ME) discussed afterwards are specified here for each eruption. n: number of opx analysed.

Opx compositions are spread over an En_46-59_ compositional range (Figs. 3a1–c5 and 4a1–d5, Supplementary Figure S2). The unzoned opx of the five eruptions have a composition between En_50-56_ (Fig. 3a1–a5). Based on the unzoned compositions and the cores compositions of the zoned opx, two populations of zoned opx have been identified (Fig. 3a1–c5): a first population (population 1) whose cores have the same composition as the majority of the unzoned opx (Fig. 4a1–b5) and a second population (population 2) whose core En contents are lower (less magnesian) than the unzoned opx (Fig. 4c1–d5). For Grand Fond, the boundary value separating the two populations is En_53_, En_54_ for Goodwill, whereas it is En_52_ for PPR1 to 3 (Figs. 3a1–c5, 4a1–d5).

*Population 1* Single-zoned crystals are mostly reverse-zoned, except for PPR1 (normal-zoned) and PPR3 (equal proportions of reverse- and normal-zoned opx) (Fig. 4a1–a5). All the multiple-zoned opx show an initial normal zoning before returning to the initial En content of the cores (Fig. 4b1–b5 and Supplementary Table S2).

*Population 2* All single-zoned crystals show a reverse pathway towards the En content domain of population 1 (Fig. 4c1–c5). For multiple-zoned crystals, different pathways are recorded, depending on the eruption (Fig. 4d1–d5). For Grand Fond, they show a reverse pathway, with their inner and outer rims in the same En domain (Fig. 4d1). For Goodwill and PPR2, they show a reverse pathway with outer rims that are richer in En than the inner ones (Fig. 4d2 and d4). For PPR1, only two multiple-zoned opx were analysed and they either show a normal or reverse pathway in the same En domain (Fig. 4d3). The multiple-zoned of PPR3, mostly record a first normal pathway from the core to the inner rims and then a reverse pathway towards the outer rims (Fig. 4d5 and Supplementary Table S2).

### Timescales

Timescales have been modelled on the most representative opx zonations. 6 diffusion profiles were modelled for Grand Fond, as well as 39 for Goodwill, 25 for PPR1, 28 for PPR2 and 35 for PPR3 (see “Material and methods”; Supplementary Figure S3 and Supplementary Table S3). 70% of Grand Fond and PPR1 profiles show the presence of high-frequency bumps and peaks on the slope or/and on the rim side of the compositional profiles, due to a growth component or unstable conditions (Supplementary Figure S3). Modelling of these crystals is not reported in the results due to uncertainty in the zonation origins^12^. Such profiles could be related to fast crystal growth kinetics prior to eruption in the magmatic reservoir, which produce overlapping and complex signals when combined with diffusive relaxation of the initial chemical gradients^44^. Furthermore, 13% of the profiles performed in Goodwill and PPR1-3 (7 for Goodwill, 3 for PPR2 and 4 for PPR3) did not show defined plateaus at the outermost edges of the crystals (Supplementary Figure S4), therefore, only 96 out of the 110 profiles analysed by EPMA in the small eruptions were used for diffusion modelling, with a temperature of 890 °C (“Material and methods”, T = 886–895 °C for these eruptions^39^). For Grand Fond, 6 profiles out of 20 were used for diffusion modelling, with a magmatic temperature of 850 °C^12,38,44^.

**Figure 5:**
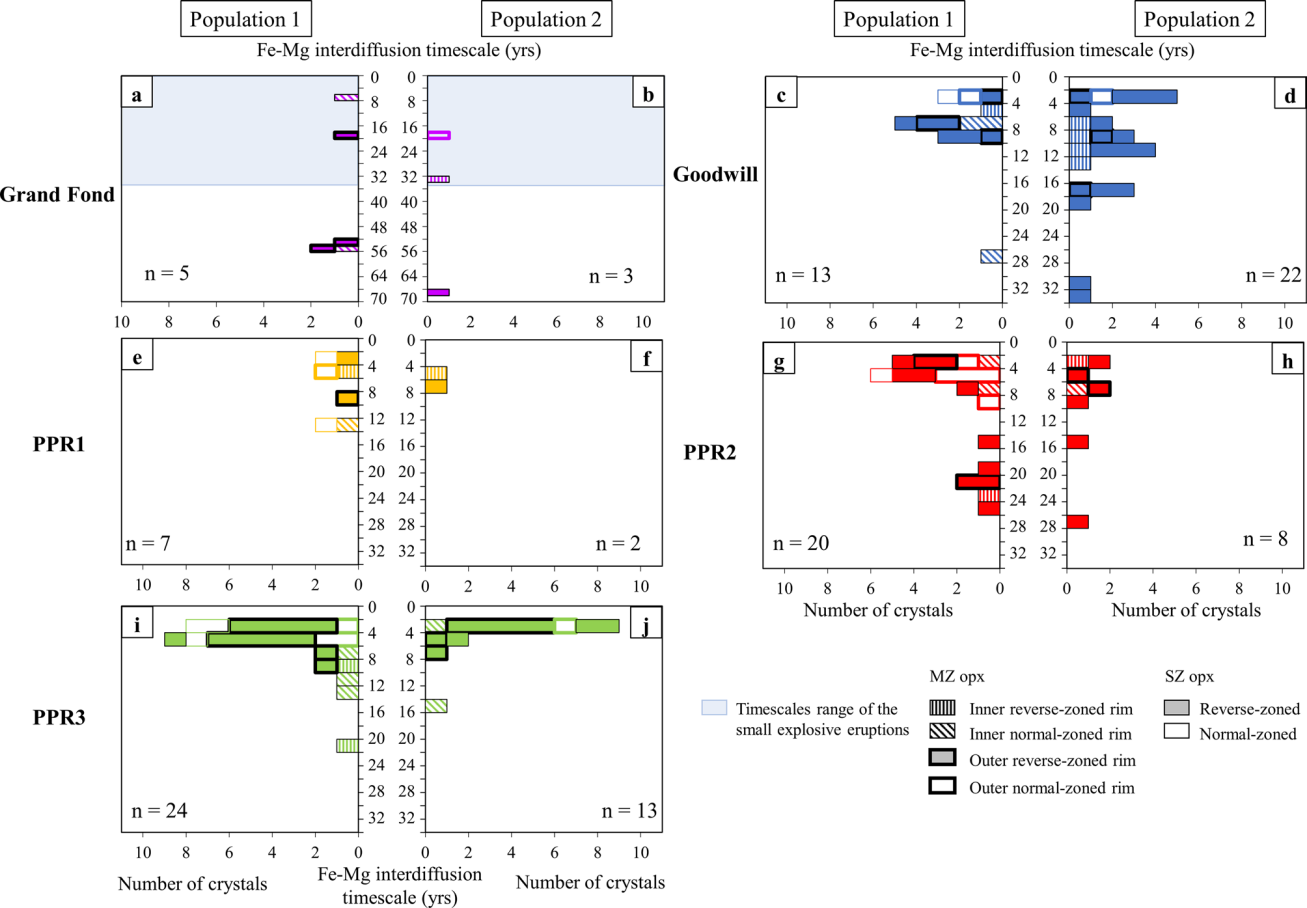
Frequency histograms of Fe–Mg interdiffusion timescales modelled in opx at 850 ± 25 °C for Grand Fond (**a**,**b**) and 890 ± 9 °C for core-rim and inner-outer rim boundaries of single-zoned (SZ) and multiple-zoned (MZ) opx of the two opx populations as in Figs. 3 and 4, of Goodwill (**c**,**d**), PPR1 (**e**,**f**), PPR2 (**g**,**h**) and PPR3 (**i**,**j**). The type of zonation is specified (reverse-zoned or normal-zoned). The timescales are presented as times before the eruptions and can be found in Supplementary Table S3. The colors are the same as in the previous figures. n is the number of profiles modelled (in SZ and MZ opx). The number “n” can be different from the total number of timescales modelled as timescales in MZ opx are sometimes modelled on both the inner and outer rims (e.g.: two timescales modelled in one profile). Individual times and their absolute uncertainties based on the propagation of a temperature uncertainty of ± 25 °C^12,38,44^ or 9 °C^39^ and uncertainties associated to SEM images are presented in Supplementary Figure S4.

For Grand Fond, the timescales are distributed between 6 and 66 years, with three timescales around 53 years (Fig. 5a,b; Supplementary Table S3). For the small explosive eruptions, across the diverse zonations considered, the timescale estimates show a large variation in both opx populations, from 1 to 32 years. Despite this, the bulk of the analyses represent the final 10 years pre-eruption (Fig. 5c–j and Supplementary Figure S5). For PPR1, timescales are between 2 to 12 years (Fig. 5e,f); Goodwill and PPR2 exhibit a wide timescale dispersion, compared to PPR1 and 3, with few single-zoned opx and some inner rims of multiple-zoned opx that give timescales longer than 20 years, but with the majority of the timescales representing the 20 years pre-eruption (Fig. 5c,d,g,h). The timescale of both inner and outer rims of multiple-zoned crystals are similar to each other and to those of the singly-zoned population. The same observation is made for PPR3, where the timescales go up to 20 years, but again the bulk of the timescales are younger than 10 years (Fig. 5i,j).

## Discussion

Considering that the proportion of zoned opx is lower in the larger Grand Fond eruption (15%) than for the four small explosive eruptions (51–65%) (Fig. 2e–i), we can conclude that these latter opx have been more comprehensively impacted by the disrupting events modifying the magmatic environments (ME—as defined in the introduction by specific storage conditions such as temperature, pressure, oxygen fugacity (*f*O_2_), volatile content^7,9–12^, see “Material and methods”). Given the proportions of zoned/unzoned opx, our results suggest that the shallow magma ponding zone feeding the recent small explosive eruptions experiences more variable changes between ME than the deep ponding zone experienced during comparable events prior to large explosive eruptions^12^. This difference could be explained by a difference in size of the reservoirs, with Grand Fond’s reservoir being larger compared to the four other eruptions, inferred by a difference in the volumes of magmas emitted^34^, as already discussed for another large explosive eruption of the same volcano, Roseau eruption^12^.

**Figure 6:**
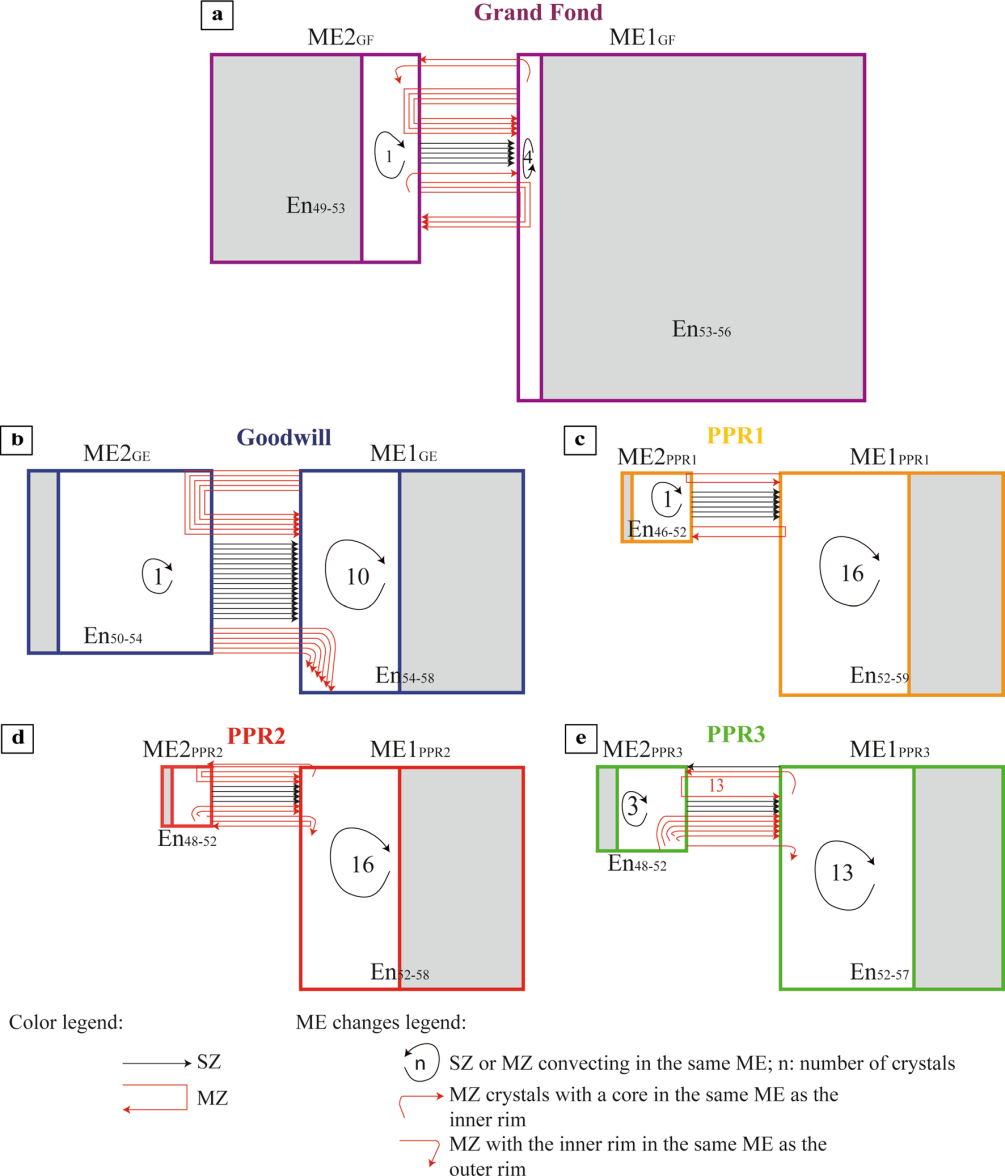
Crystal System Analysis approach of the zonations recorded by the two populations of opx of the five eruptions studied [Grand Fond (**a**), Goodwill (**b**), PPR1 (**c**), PPR2 (**d**) and PPR3 (**e**)]. Each box represents a magmatic environment (ME): ME1_GF_ = En_53-56,_ ME2_GF_ = En_49-53_; ME1_GE_ = En_54-58,_ ME2_GE_ = En_50-54;_ ME1_PPR1_ = En_52-59,_ ME2_PPR1_ = En_46-52_; ME1_PPR2_ = En_52-58,_ ME2_PPR2_ = En_48-52_; ME1_PPR3_ = En_52-57,_ ME2_PPR3_ = En_48-52,_ for Grand Fond, Goodwill, PPR1, 2 and 3, respectively. For each eruption, the size of the boxes is approximately proportional to the proportions of unzoned and cores of zoned opx in each ME, using the volumes of ME calculated in Supplementary Table S4. The grey zones show the proportions of unzoned crystals and the white areas, the proportions of zoned crystals (reset to 100%, Supplementary Table S4). The change of ME recorded from the core to the rim of opx is represented with a black arrow for single-zoned (SZ) crystals and a red arrow for multiple-zoned (MZ) crystals. For PPR3 (**e**), 13 multiple-zoned crystals record a change from ME1_PPR3_ to ME2_PPR3_ (13 in red). In the case of the studied magmas, an En increase (reverse-zoned) implies a temperature increase, with, or without, magma mixing with a more basic and hotter magma. Conversely, an En decrease (normal-zoned) may be linked to a pressure drop, decrease in melt H_2_O content or mixing with a colder magma^38^. This Figure has been drawn using Adobe Illustrator (Version 24.0.1; https://www.adobe.com/fr/products/illustrator.html).

Based on the proportions of zoned opx in the five eruptions (Fig. 2), on the proportions of unzoned and cores of zoned opx and their En content (Figs. 3, 4), we may define two ME (named ME1 and ME2). We may estimate the relative size of the two ME that can be representative, for Grand Fond, of the volume of the two batches of magma containing respectively ME1 and ME2 (Supplementary Table S4). For Grand Fond, the two ME are ME1 (En_53-56_) and ME2 (En_49-53_) (Figs. 3a1,b1,c1 and 4a1–d1). ME1 contains ~ 62% of the opx crystals against ~ 38% for ME2 (Supplementary Table S4). Provided that the crystallinity of the two ME is similar, this would suggest that ME1 is around twice as large than ME2. As with the two other large explosive eruptions studied previously^12^ (Roseau from the same volcano and Layou from Morne Diablotins)^12^, reverse-zoned crystals are dominant (88% of the single-zoned crystals, Fig. 2). ME1 contains most of the unzoned opx (59%), few single-zoned cores and multiple-zoned opx (Supplementary Table S4, Fig. 6a). Some multiple-zoned ones have a core and an outer rim in ME1 whereas others have an inner rim in ME1 (for core and outer rim in ME2). ME2 is represented by fewer unzoned opx (26% of the crystals, Supplementary Table S4), cores of single-zoned and multiple-zoned crystals (Supplementary Table S4, Fig. 6a). Some multiple-zoned crystals with cores in ME1 have inner rims in ME2 whereas others have their cores and outer rims in ME2 (and inner rims in ME1) (Fig. 6a). The low variation of En content between these two ME indicates a low temperature difference between them (~ 20 °C, considering an En/T relationship based on experimental data^38^). It is then likely that these ME were probably located at relatively similar pressure/depths.

For the four recent and small explosive eruptions, opx globally tell us the same kind of story (Fig. 6b–e), with two ME identified for all these eruptions. The relative sizes of the different ME are more or less similar (except for Goodwill, that has a bigger ME2 than for PPR1-3, Supplementary Table S4, Fig. 6b–e), as well as the proportions of zoned and unzoned crystals. Most of the single-zoned crystals have a core in ME2 and a rim in ME1 and, for most of the multiple-zoned crystals, a core and outer rim in ME1 and an inner one in ME2. A large proportion of zoned crystals show low variations of En in the same ME (ME1), they are probably convecting in the same ME. Changes in En content between cores and rims of opx can be interpreted as an evidence of magmatic processes taking place in magmatic reservoirs such as convection, sedimentation, edge heating or cooling, volatile transfer^4,45–47^ leading to changes in crystallization conditions^48^. Following the CSA approach, zoned opx have experienced different ME during magma residence, creating zonations. The CSA diagrams of these four small explosive eruptions illustrate a repeating system with similar or nearly identical behaviors recording a global increase of the temperature (Fig. 6b–e). For these eruptions, the low variation of En content in the opx between the two ME indicates that the two batches of magma containing the two ME are probably located at the same depth in the transcrustal system of Morne Trois Pitons-Micotrin.

Melt inclusions (MI) and matrix glasses have been extensively analyzed in opx^39,41^ (Supplementary Figures S6–8; Supplementary Table S5). For all eruptions, MI were abundant and of significant sizes (20–50 µm^39^) in the cores of the opx but less frequent in the rims and/or too small in size to be analyzed (< 5 µm). MI hosted in opx cores continuously cover a range of composition from 75 to 79% in SiO_2_ and 1–2.5 wt% in CaO (Supplementary Figure S8). These small variations in composition do not show significant differences between the two ME (Supplementary Figure S6). These MI were also volatile-saturated^39^. Some matrix glasses were also analyzed showing, for all the eruptions, a SiO_2_ content between 75 and 77 wt% comparable to the less silica-rich MI (Supplementary Figures S7–8). The data on major element glass composition (Supplementary Figures S7–8), supports the idea that no basic magma or magma with a significant different composition was involved in the pre-eruptive dynamics of the eruptions. More likely, the ME of this study partook of mixing with magmas of close compositions but possibly different temperatures, to explain the observed crystal zonations and the compositions range of the MI, suggesting a thermally zoned reservoir, with reservoir walls corresponding to a lower temperature environment than its core.

The question then arises of the origin of the unzoned opx (85% of the opx are unzoned for Grand Fond and 35–49% for the four smaller eruptions, Fig. 2). Three possible interpretations are considered. Firstly, unzoned crystals could represent a zero-age population that grew after the mixing event that formed the rims in the zoned crystals. This might be unlikely assuming similar growth rates as unzoned opx should be of similar age as the zoned opx based on their size. Secondly, unzoned crystals might be old crystals that had enough time to fully or near-fully re-equilibrate, but it would take longer than the probable gap between eruptions. Finally, unzoned crystals were present in the magmatic reservoir but in a part of the reservoir that wasn’t affected by the mixing process so they do not record that event.

As the majority of the unzoned opx are part of ME1 for all the eruptions, we suggest that they were present before the mixing event. For the unzoned opx with a core composition of ME2, we consider that they represent opx from this particular ME that got mixed with ME1 without recording any changes in compositions. This is particularly true for Grand Fond, with a larger reservoir inferred in which the mixing process was more limited. To better address this question, examining the Al content can be useful as it diffuses slowly in opx and will reflect growth and recrystallisation processes with only a minor diffusion overprint^25,43,49^. Comparison of the Al_2_O_3_ content relative to En content for the unzoned and zoned opx has been undertaken for all eruptions (Supplementary Figure S9). In all cases, unzoned opx are in the same compositional domain in Al_2_O_3_ (wt%) as the majority of the cores of zoned opx (between 0.4 and 0.8 wt%). Their En content also overlaps the core compositions of the zoned opx. Thus, they were probably in a part of the same source reservoir but a part that never participated in the mixing process. The unzoned phenocrysts are then likely to be representative of the magma state prior to any perturbation (as a mixing event) investigated for diffusion modelling.

**Figure 7:**
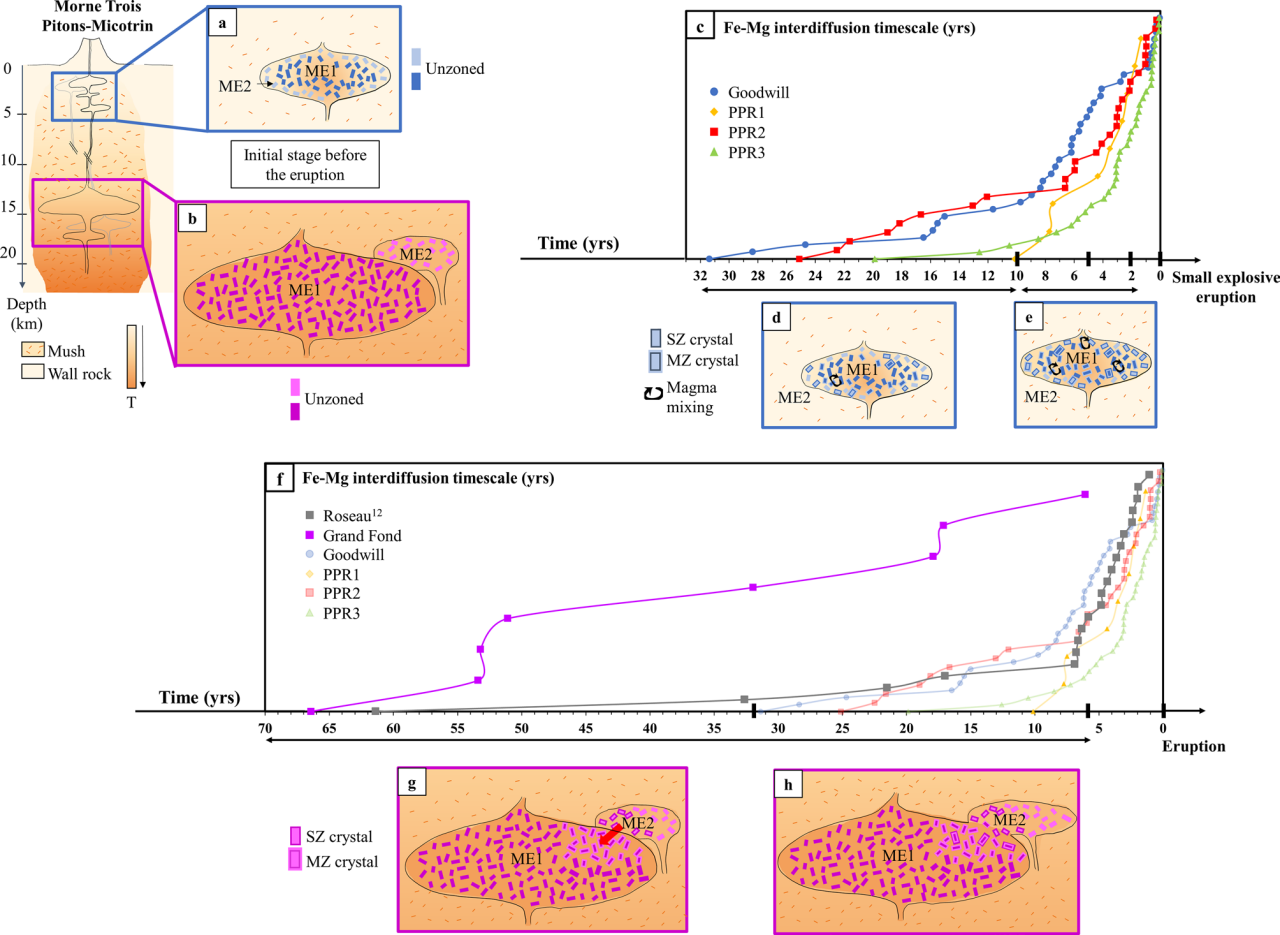
Simplified view of the storage areas and of the pre-eruptive dynamics of the magmas in the magmatic mush for Morne Trois Pitons-Micotrin. Models based on opx compositions and interdiffusion timescales modelling for the small explosive eruptions and Grand Fond. The different magma environments recorded are represented by magma lenses at different depths in a transcrustal system. This schematization agrees with the vision of the magmatic mush showing magma lenses stored at different depths^6^. On top: model of the whole crust with a deep reservoir feeding the large explosive eruptions and a shallower one feeding the small explosive eruptions^38,39^. (**a**,**b**) initial stages for small explosive eruptions (Goodwill, PPR1-3) and voluminous pumiceous eruption of Grand Fond; **c**: rank ordering of the interdiffusion timescales modelled in the opx of Goodwill, PPR1-3 (Fig. 5; Supplementary Table S3). Each curve in (**c**) can be divided into three gradients (underlined by black arrows): a shallow one (from 32 to 10 years), a steep one (from ~ 10 to 2–3 years), together with that of a pre-eruptive region (timescales from 0 to 2-years, vertical arrow). A steep gradient can indicate a rapid processing whereas a shallow gradient, a much slower processing. (**d**,**e**) evolution of the magmas prior to the small explosive eruptions with vigorous magma mixing in a temperature-zoned reservoir, with ME2 being the wall and ME1 the center of the reservoir, in the different periods of time underlined in (**c**); (**f**) rank ordering of the interdiffusion timescales modelled in Grand Fond and Roseau^12^, large explosive eruptions from Morne Trois-Pitons Micotrin volcano compared to the small explosive eruptions (Fig. 5; Supplementary Table S3). In (**f**), for Grand Fond, one gradient can be identified (from 66 to 5 years). For Roseau, two gradients can be identified: a shallow one (from 60 to 7 years) and a steeper one from 7 to 1 year before the eruption. (**g**,**h**) evolution of the magmas prior to the large explosive eruption with magma mixing following the period of times shown in (**f**). T: temperature; black arrow: magma mixing. SZ: single-zoned opx and MZ: multiple-zoned opx. This Figure has been drawn using Microsoft Office suite 2019 Version 1808 (https://www.microsoft.com/fr-fr/microsoft-365).

The configuration of the magma plumbing system beneath Dominica inferred by previous studies is that of a transcrustal system with batches of magma stored at different depths (~ 12–16 km for the large explosive eruptions and ~ 2–8 km for the small explosive eruptions^38,39^, Fig. 7a,b). Considering these different storage depths, the magmas represented by the different ME are probably stored in a mush. For Grand Fond, as ME2 is also recorded by a large number of opx, we suggest that two batches of magma ME1 and ME2 interacted (Fig. 7b), as for Roseau^12^. They are probably located at the same depth considering the low En variation content in the opx. The large proportion of unzoned crystals (85%) and their abundance in the most voluminous ME1 indicated that a limited mixing process occurred (Fig. 7g,h). For this eruption, the two magmas may probably be stored in two different close reservoirs showing a small composition and temperature difference; the eruption is linked to the injection of magma from ME2 (the smallest reservoir) into ME1, the more voluminous reservoir. The same scenario was described for the large explosive eruptions of Roseau and also for Layou^12^ from Morne Diablotins volcano.

For all the small explosive eruptions, the mixing was more efficient, as a larger proportion of crystals are zoned. The volume of magma mobilized for each eruption is lower than for the large explosive eruptions. All these eruptions show the same scenario with proportions of magma from ME1 and ME2 in the same order of magnitude (slightly higher for ME2 in Goodwill). The small En difference between ME1 and ME2 indicates a small difference of temperature and consequently of depth between the two magmas. Considering that (i) a small volume of magma is mobilized for each eruption (less than 1 km^3^), (ii) the magmas from the two ME have a small composition and temperature difference and are stored at the same depth (shallower than the magma reservoirs of Grand Fond), (iii) the same scenario occurs for all these eruptions in less than 10 kyrs and (iv) compositions of the MI are close and volatile saturation was reached in magmas prior eruption, it is difficult to propose two distinct reservoirs for these eruptions. The most reliable hypothesis is the presence of a thermally zoned reservoir which is not totally emptied after each eruption (Fig. 7a). Between the eruptions, a classical magma evolution within the reservoir would lead to the formation of the two ME, the high-temperature ME1 in the center and low-temperature ME2 being located at the wall of the reservoir. The two magma ponding zones may be linked by replenishment of the shallower one by deeper magmas, which possibly explains the strong similarities in composition between all magmas, except for the volatile content^39,41^ (see Supplementary Material).

To further investigate the dynamics of the plumbing system, timescales modelled by intracrystalline diffusion can be coupled with CSA. For Goodwill, as the compositions of the multiple-zoned inner and outer rims are very close, and it is likely that the crystallization of the inner and then outer rims occurred closely in time and could reflect different aspects of the same mixing event. This assumption is verified by the timescales modelled here: timescales modelled on the same multiple-zoned opx for both the inner and outer rims are approximately the same (from 1 to 10 years, Supplementary Table S3 and also for one opx of Grand Fond). Furthermore, for all the small explosive eruptions (Goodwill and PPR1-3), there are no systematic time differences between the inner and outer rims of other multiple-zoned opx and the rims of single-zoned crystals and a greater proportion of them lie within the range 1–10 years. Exceptionally, some rare single-zoned opx give timescales older than 10 years, up to 32 years (Supplementary Table S3).

This could imply that the mixing process evolves through time from single and multiple exchanges, for the small eruptions, 10–32 years before the eruption, to something far more dynamic near the time of eruption (~ 2–10 years) and before the eruption (timescales of ~ 1-year, Fig. 5c–h). For Grand Fond, longer timescales have been obtained (based on the eight timescales modelled on profiles without bumps, Fig. 5a,b; Supplementary Table S3), the remobilization of the magma ponding zone then possibly occurred several decades prior to eruption (up to 66 years before). These timescales estimates highlight a gradual reactivation without an acceleration of the exchanges prior to the eruption.

For Roseau, another large explosive eruption (33 kyrs) in Dominica from the same volcano, presenting the same parameters in terms of storage depth, magma temperature, pressure, *f*O_2_, shapes and values of the opx zonations, a sustained magma mixing phase has been inferred, 3 to 10 years prior to eruption, after a reactivation period of several decades^12^. As Roseau, some timescales in Grand Fond are also longer than 30 years. The timescales characterizing the processes then seem to be close for both the small explosive eruptions and the larger ones, with longer timescales recorded for Grand Fond.

To decipher the spatiotemporal dynamics in the reservoir, rank ordering diagrams of the timescales modelled for each eruption have been made; curved ramps are associated to each eruption (Fig. 7c,f). These curves can be divided into three gradients for the small explosive eruptions, going from the older timescales to the younger ones with a shallow gradient, then a steeper one and a short vertical one (young population of timescales around 1–2 years) (Fig. 7c). A steep gradient shows a rapid processing whereas a shallow one, a much slower processing. The steep regions of PPR1-3 are much steeper than Goodwill (Fig. 7c); their processing is therefore faster. PPR1’s curve only seems to have a steep gradient but this could be biased due to the lower number of crystals studied. This suggests a rapid build up to eruption over a decade at a sustained, relatively high, rate. PPR2 interactions between magmas started about 25 years ahead of the eruption, transitioning to a rapid process some 2–7 years prior to the eruption (Fig. 7c). PPR3 started with a similar slow build up about 20 years pre-eruption, then moved to the rapid process 5 years pre-eruption (Fig. 7c). This all contrasts a bit with Goodwill, which followed a trajectory close to PPR2 but with gradual slopes, and then seems to have stopped about 3 years pre-eruption before suddenly moving to eruption (Fig. 7c).

For Grand Fond, contrary to the other eruptions, Grand Fond’s curve seems to be defined by only one gradient: from ~ 70 to 5 years (Fig. 7f). Timescales modelled on bumps go from 1 to ~ 170 years (Supplementary Figure S10, Supplementary Table S3). We conclude that a continuous reactivation is found for Grand Fond, with no rapid build-up in the 10 years pre-eruption as found for the small explosive eruptions.

In comparison, Roseau curve follows the same trajectory as Goodwill, with a slower build up (from 60 years), to a fast one 7 years prior to the eruption^12^ (Fig. 7f).

During the decades preceding the small eruptions, some interactions between the two magmas occur and progressively increase up to the last 2 years before the eruptions, where the mixing is more efficient, marked by an increase in the mobilisation rate 2–10 years before all eruptions apart from PPR1 (Fig. 7c), and would gradually leads to overpressure in the reservoir and to the eruption (Fig. 7d,e). Thus, a 10-year warning might be observable e.g., increased seismicity, inflation, degassing and a geochemical response of the hydrothermal system near the Morne Trois Pitons-Micotrin volcano (Valley of Desolation-Boiling Lake system^50^). These signals could be detectable using seismic data or remote sensing techniques, which is the subject of ongoing research. It is necessary that, during the time separating the eruptions, magmas with a similar composition and probably stored in different pockets in the same order of depth are reinjected in the main reservoir.

Parallel study of plagioclase crystals^38,39^ erupted by these five eruptions leads to results consistent with the opx study, with significant development of sieve textures typical of mixing with hotter magma and crystal dissolution (Supplementary Figure S11).

We can then propose a spatiotemporal evolution of the magma plumbing system within a transcrustal system, beneath Morne Trois Pitons-Micotrin volcano in Dominica, using opx as they are trackers of pre-eruptive processes (Fig. 7). Two main magma storage regions are present at Dominica, one at 12–16 km^38^ and another at 2–8 km^39^, with variable interactions between batches of magmas through time. 33 kyrs ago, the deeper system saw partial mixing between two magma batches one decade or more prior to the voluminous pumiceous eruptions of Roseau. The eruption of Grand Fond then followed, 9 kyrs later, with similar behaviours but without a dynamic reactivation shortly before the eruption. Since 18 kyrs, magma storage areas, lower in volume, formed at a much shallower depth (~ 2–8 km^39^), with lower volumes of magma emitted during the successive eruptions of Goodwill Entrance, and PPR1 to PPR3. These eruptions are characterized by vigorous and relatively extensive mixing in a thermally-zoned reservoir, possibly following an injection of a hot magma from a deeper source. These magmatic zones are likely to have been disturbed ~ 10 to ~ 30 years prior to eruption and then more dynamically ~ 2–10 years prior eruption, with activity ramping up prior to the resulting eruption (Fig. 7). Such investigations have a potentially major role in terms of volcanic risk mitigation and management of future explosive volcanic crises.

## Material and methods

### Sampling

The studied samples are pumices which were collected during field campaigns in Dominica between 2011 and 2013 and were at the base of previous studies^12,34,38,39,41,44^.

### Sample preparation

The unaltered pumice samples were crushed (up to 3 mm). One half was then crushed again into fine powder for whole rock analysis while the other half was sieved into different size fractions (1 mm–750 µm, 750–500 µm, 500–355 µm, 355–250 µm, 250–125 µm). These fractions have been washed in ultrasonic bath and dried at 80 °C for 48 h. They were then observed under the binocular microscope to select the fractions in which the crystals were the more abundant and automorphic. Opx were then handpicked under the binocular microscope from size fractions ranging from 125 to 500 µm. They were mounted in epoxy resin with c-axis in a north–south direction as they have been used for intracrystalline diffusion modelling and polished up to 0.3 µm to the middle part of the opx.

3224 automorphic orthopyroxene (opx) crystals randomly chosen were studied for the five eruptions. Major and trace element studies were performed on opx (Supplementary Figures S2, S6–8). In Grand Fond, 814 opx were studied in the 355–500 µm fraction. In the four small explosive eruptions, 2410 opx have been observed, including 1100 opx in the 355–500 µm fraction, 840 in the 250–355 µm fraction and 470 in the 125–250 µm fraction. The proportions of zoned opx are very similar whatever the considered fraction (Supplementary Figures S12–14). Thus, here, the three fractions are gathered and discussed simultaneously.

Plagioclases (plag) and magnetites (mgt) were also handpicked and prepared for textural observations and analyses. Before Scanning Electron Microscope (SEM) or electron microprobe micro-analyzers (EPMA) investigations, selected mounts were all carbon-coated.

### Textural observations: scanning electron microscope

All the crystals were observed by using a Scanning Electron Microscope (SEM), Zeiss Supra 55VP (Sorbonne Université, Paris). To identify zoned and unzoned crystals, back-scattered electron (BSE) images were systematically acquired. Proportions of zoned/unzoned crystals have been determined on all the crystals mounted by looking closely at the SEM images. On zoned crystals, high resolution BSE images were acquired on selected chemical zonations with 20 kV acceleration voltage, a beam current of 8 nA and high integration line (8 integrations per line to reduce the noise) with a dwell time per pixel of 92 µs. These high-resolution BSE images were used for the diffusion modelling and intercalibration with chemical profiles.

### Compositional analysis of crystals and melt inclusions by EPMA

Opx crystals have been analysed for major elements (Si, Na, Ti, Al, Ca, K, Fe, Mg, Mn, P, Cl) with an acceleration voltage of 15 kV, a beam current of 10 nA and a focused beam (CAMECA SX-Five and SX-100; Service Camparis, Paris). Counting time on peak and background for Fe and Mg was set at 80 s and at 10 s for the other elements. The core to rim compositional profiles in zoned opx had a 2 µm step and an average length of 100 µm (~ 4 h per profile). Four points were measured across the unzoned crystals. They were acquired perpendicular to the long axes of the opx and away from the corners to avoid three-dimensional effects such as growth^25,42,43^ (Fig. 2b–d).

Glassy melt inclusions in opx and matrix glass were also analysed by EPMA for major elements with the same conditions as described in other studies on Dominica^39,41^. An acceleration voltage of 15 kV was used, with a beam current of 10 nA. Counting time was set at 10 s for the major elements. Na was first analyzed to minimize loss by volatilization under the electron beam^39^. All precautions required for MI investigations were applied: only glassy crystal- and bubble-free ones with no post-entrapment modifications have been investigated^39,41^.

### Deciphering spatiotemporal magma dynamics: coupling the crystal system analysis and Fe–Mg interdiffusion modelling of timescales

Stable storage conditions define a given magmatic environment (ME) and are recorded during the growth of the crystals. If the conditions change, the modification of ME is recorded by forming chemical zonations (Supplementary Figure S15) by crystallization, these ones are then equilibrated by diffusion of chemical elements.

A Crystal System analysis (CSA) approach has been developed before on olivines^7,9–11^, according to previous works of this approach^8^. The CSA is the systematic analysis of the crystal compositions to investigate in time and space the changes of crystallization conditions. It is a schematic view of the crystal evolution in different ME that permits to study the conditions of crystal growth from the core to the rim. Each ME is represented by a box and arrows between them indicate the passage, from the core to the rim, of the recorded environments for single-zoned or multiple-zoned crystals. The main path(s) between the ME can then be identified statistically. Here, we applied the CSA approach to the opx^12^. Opx are interesting mineral phases as their composition is sensitive to temperature and thus may help us to track the pre-eruptive dynamics of the plumbing system^38,51^. Compositional plateaus in zoned opx crystals can be identified, with EPMA, as zones of at least 10 µm length where the enstatite content (En content; $\frac{\text{Mg }}{\text{Mg }+\text{ Fe}}$) is constant with a variability of ± 0.5%. If the same compositional plateau is found in a significant amount of crystals, these plateaus have been produced by significative changes in the magmatic conditions^7,52^ and can be considered as ME (Supplementary Figure S15). An En increase (reverse-zoned opx) implies a temperature increase, with, or without, magma mixing with a hotter and more basic magma. Conversely, a decrease in the En content (normal-zoned opx) may be linked to a pressure drop, decrease in melt H_2_O content or mixing with a colder magma^38^.

Here, we have coupled the CSA to the interdiffusion Fe–Mg modelling to estimate the timescales of the pre-eruptive history.

The rate of diffusion of chemical species in the crystal lattice has a strong role on the preservation or resetting of compositional zoning during magma storage at high temperature^52–54^. The diffusion coefficient depends on parameters such as chemical composition (Xi; molar fraction of the mineral constituent element), temperature (T in Kelvin), pressure (P in Pa), oxygen fugacity (*f*O_2_) and water fugacity (*f*H_2_O)^21,29^. Thus, by knowing the diffusion coefficient and by constraining the chemical composition, it is possible to determine Fe–Mg interdiffusion timescales associated to a change in the magmatic conditions that created the zonations in the crystals.

Modelling of the timescales associated to the zonations in the zoned crystals has been done by using the method developed in other studies^25,55^, using the parametrization of the interdiffusion coefficient of Fe and Mg elements in opx^56^, D, which depends on the composition of the opx^12,21^. The Fe–Mg interdiffusion profiles were modelled in one dimension, across the c-axis, parallel to the b-axis of the crystals^25,55^. The oxygen fugacity is an important parameter, though its influence on the Fe and Mg diffusion coefficient is still under discussion. Previous studies have shown that when integrated in the diffusion coefficient parametrization, differences in the timescales modelled appear. For example, timescales modelling on opx of two Santorini eruptive units give timescales 30–40 times greater than when using another coefficient^25,43,57^.

If the same mechanism of vacancy generation through iron oxidation is taking place, it has been conjectured that the *f*O_2_ dependence could follow the same form as that for olivine (with an exponent of 1/6)^12,56^. This equation parameterization seems to be in accordance with experimental results on opx with Mg# superior to 0.90^57^, arguing for a minor dependence of the diffusion coefficient on *f*O_2_ (with an exponent of 1/20 instead of 1/6)^12^. However, this study was conducted on pyroxene very close to En end member^57^, which is not in adequation for the samples studied here (En_46-59_).

The correction of the diffusion coefficient for *f*O_2_ made in the previous study^25^ seems to overestimate the *f*O_2_ dependence compared to the other diffusion coefficient parametrization^57^, making calculated diffusivities about one order of magnitude higher than the measured values under common geological conditions (close to NNO buffer)^25,44,57^.

Another study in iron rich orthopyroxenes (for temperatures between 1050 and 1200 °C and at Quartz-Fayalite-Magnetite oxygen buffer) reported a diffusivity of D_Fe-Mg_ = 3.10^−19^ m^2^.s^−1^ at T = 1130 °C^12,58^. If the results of the first authors^56^ are extrapolated to higher temperatures, they are in good agreement with those of made in the iron rich orthopyroxenes study^12,58^. For these reasons, the oxygen fugacity will not be considered. The expression of the diffusion coefficient we used is valid for a temperature between 500 and 800 °C, an oxygen fugacity of WI at WI + 0.8 log units above the Iron-Wustite buffer (WI: Iron-Wustite) and X_Fe_ = 0.10–0.50, molar fraction of the ferrosilite component^56^. The En range of the opx is En_46-59_ (X_Fe_ = 0.45–0.52), which is in the stated calibration range of the parametrization^56^ (Supplementary Figure S2). For Grand Fond, the estimated pre-eruptive temperature is 850 ± 25 °C^38^. The estimated pre-eruptive temperature of the opx of the four small explosive eruptions is about 890 °C (with an uncertainty of ± 9 °C)^39,59^, which is slightly above the range of used temperatures in the method^21^. Thus, for our diffusion model, the values of the coefficient D used for each profile extrapolate the diffusion coefficient parametrization^56^ and are deduced for a temperature of 850 or 890 °C and a given chemical composition (maximum and minimum D calculated by the model using the maximum and minimum En contents of the profile considered).

The initial conditions used for this model are a step-wise profile^12^. Over time, Fe–Mg interdiffusion tends to homogenize the chemical gradient, induced by changes in magmatic conditions, the zonation is then smoothed and the chemical profile is turned into a sigmoid. Since the Fe–Mg interdiffusion is frozen during the eruption, depending on the shape of the sigmoid observed on the profile, it is possible to determine diffusion timescales corresponding to these compositional changes.

The model consists in intercalibrating chemical composition profiles of zoned opx (expressed by the Mg number, $\text{Mg}\#=\frac{\text{Mg}}{\text{Mg}+{\text{Fe}}_{\text{tot}}}$) obtained with an electron microprobe with grayscale profiles on high-resolution north–south oriented images taken with SEM (spatial resolution of 755 nm), as the Mg number is the parameter that seems to best control the grayscale of the images^44,55^. These grayscale profiles are produced with the *ImageJ* software (https://imagej.nih.gov/ij/; version 1.52a) and are averaged over a certain width to reduce the noise (between 20 to 50 µm). Following this intercalibration, the profiles are modelled to obtain a time constraint between the possible perturbation recorded by the crystals and the eruption. A database of contrasts of particular compositions is available in our model, the curve best corresponding to the one obtained by intercalibration will be searched in this database^55^. A rescaling between the two curves will have to be performed. This scaling factor depends on several parameters: the ratio between the timescales of the database and the time associated with zoning in the crystal which allows to find the timescale corresponding to the zonation^12^.

The uncertainties associated to the timescales modelled come from the determination of the temperature, the diffusion coefficient and its measurement^19,21^. The accuracy of the measuring instruments used to model the diffusion timescales are also sources of uncertainties: SEM images and electron microprobe profiles (uncertainties on the calibration of measurements, point spacing), as well as the resolution of SEM images. However, the largest sources of uncertainty would come from the measurement of the diffusion coefficient and temperature^12,21,44^ (Supplementary Figure S5).

## Supplementary Information

Supplementary Information 1.

Supplementary Information 2.

Supplementary Information 3.
