# Supplementary material for: Time-window into the transcrustal plumbing system dynamics of Dominica (Lesser Antilles)
Source: Sci Rep. 2021 Jun 1;11:11440. doi: 10.1038/s41598-021-90831-1 (PMC8169881; doi:10.1038/s41598-021-90831-1)
Supplement: Supplementary file 1 — Supplementary Information 1. [file 41598_2021_90831_MOESM1_ESM.pdf]

Time-window into the transcrustal plumbing system dynamics of Dominica (Lesser Antilles)

Lea Ostorero[1], Georges Boudon[1], Hélène Balcone-Boissard[2], Daniel J. Morgan[3], Thiebaut d'Augustin[2], Clara Solaro[1]*

[1] Université de Paris, Institut de physique du globe de Paris, CNRS, F-75005 Paris, France

[2] Institut des Sciences de la Terre de Paris (ISTeP), UMR 7193, CNRS-Sorbonne Université, Paris, France

[3] Institute of Geophysics and Tectonics, School of Earth & Environment, University of Leeds, Leeds LS2 9JT, United Kingdom

Corresponding author: Léa Ostorero (ostorero@ipgp.fr)

Supplementary material

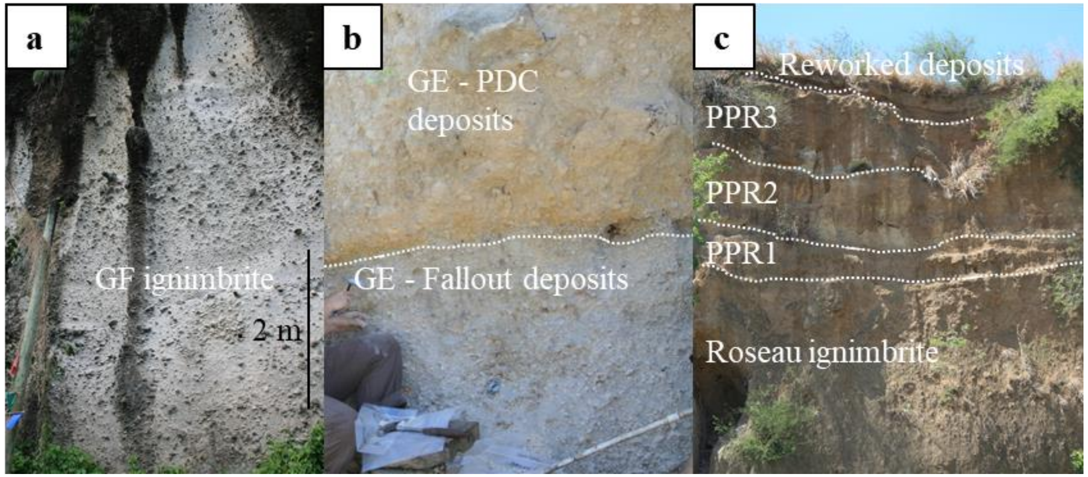

Supplementary Figure S1: Pictures of the outcrops of Grand Fond (GF), Goodwill (GE) and PPR1 to 3 in Dominica: a. Grand Fond pyroclastic density current; **b.** Goodwill fallout deposit; **c.** PPR1, 2 and 3 fallout deposits on top of the Roseau ignimbritic deposits.

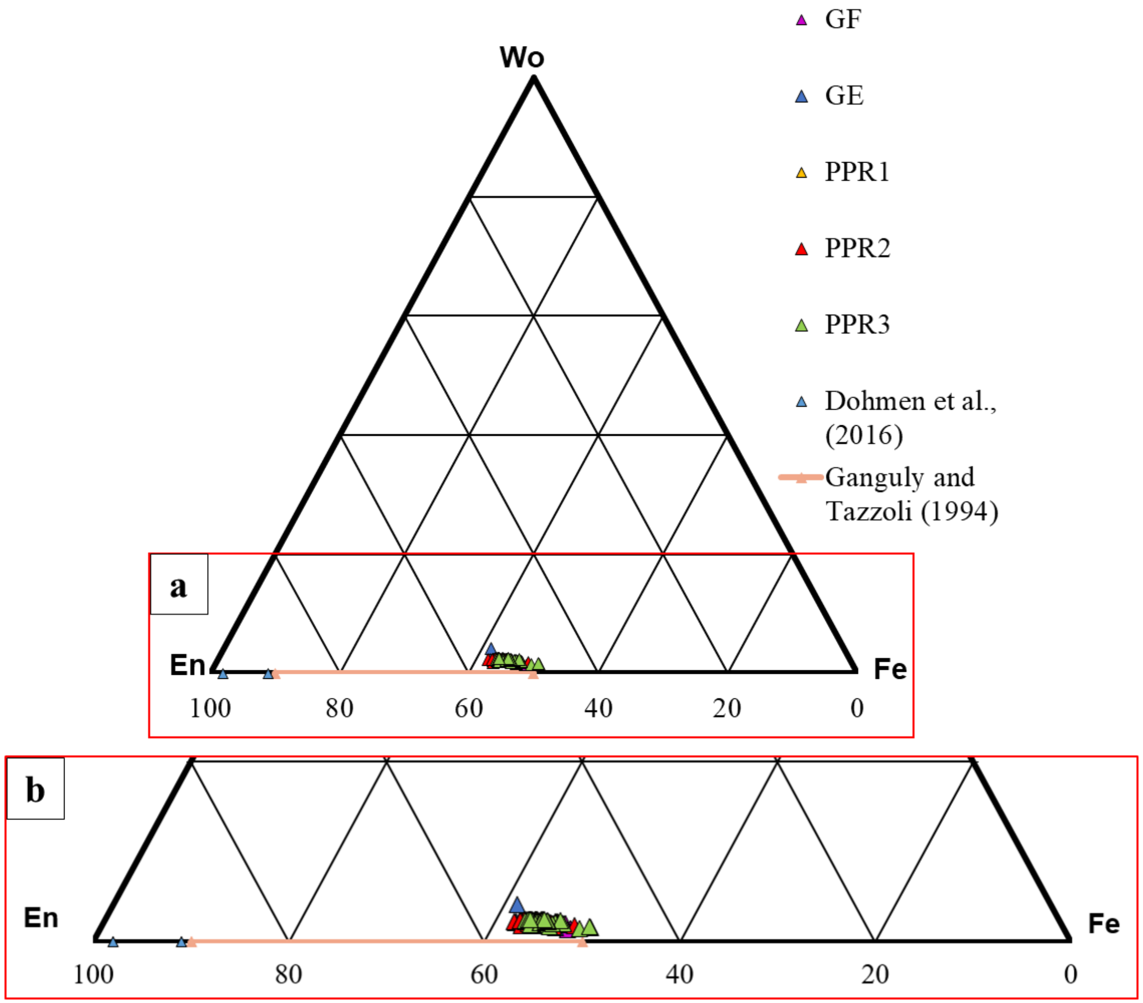

Supplementary Figure S2: Ternary diagram of the unzoned opx compositions of Grand Fond (GF), Goodwill (GE) and PPR1-3. a. Ternary diagram with the three poles (Wollastonite (Wo), Enstatite (En) and Ferrosilite (Fs)); **b**. Zoom on the opx compositions. The colors of the eruptions

are the same as in the main article (**Figure 2-4**). In orange: En contents domain for which the Fe-Mg interdiffusion coefficient parametrization is formulated[1] and other experiments conducted close to the Mg endmember[2]. Note that En_{46} is just outside the stated calibration range of the interdiffusion coefficient[1].

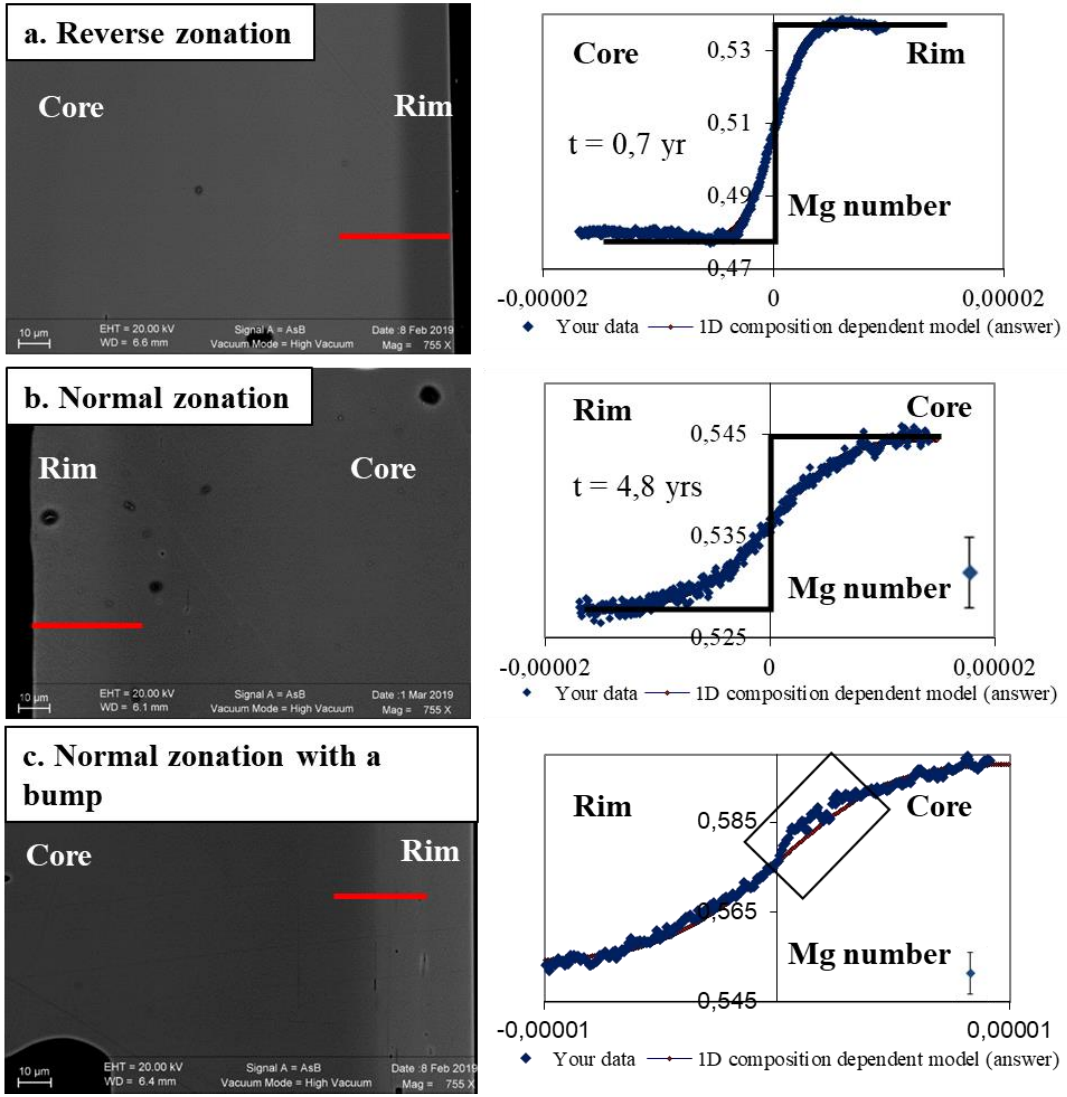

Supplementary Figure S3: High resolution Scanning Electron microscope (SEM) images and modelled diffusion profiles for three orthopyroxenes (opx) of our opx set having a reverse (a), a normal zonation (b) and a normal zonation (c) with a bump due to growth near the core in an opx of the PPR1 eruption. The red line shows the location of the electron microprobe profile (EPMA). The profiles represent the Mg number as a function of the distance from the core (µm). The blue curve represents the intercalibration of the electron microprobe and greyscale data whereas the black curve is the 1D composition dependent model that best fits the blue curve. The associated timescales corresponding to the fit are specified on the graphs. The black step represents the initial conditions: a step function and shows the profile as it would have been if the

zonation had not been rebalanced by the Fe-Mg interdiffusion. The black rectangle in (**c**) represents a portion of the profile affected by the presence of a bump.

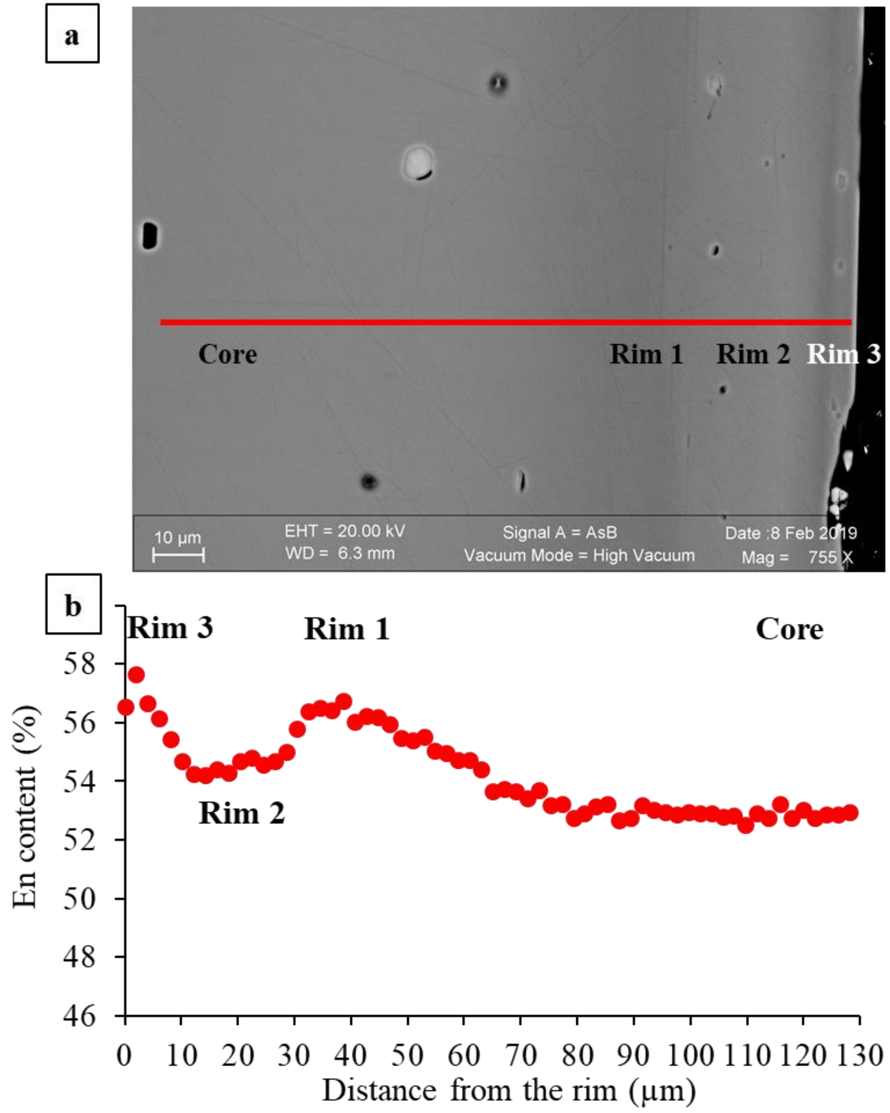

Supplementary Figure S4: Example of a multiple-zoned orthopyroxene, displaying a third rim measuring less than 10 µm and then not considered as a plateau in the results. a. SEM high-resolution image with the red line representing the location of the EPMA profile; **b. Associated EPMA profile in En content (%) with three major changes in the En content with the last one measuring 2 µm.**

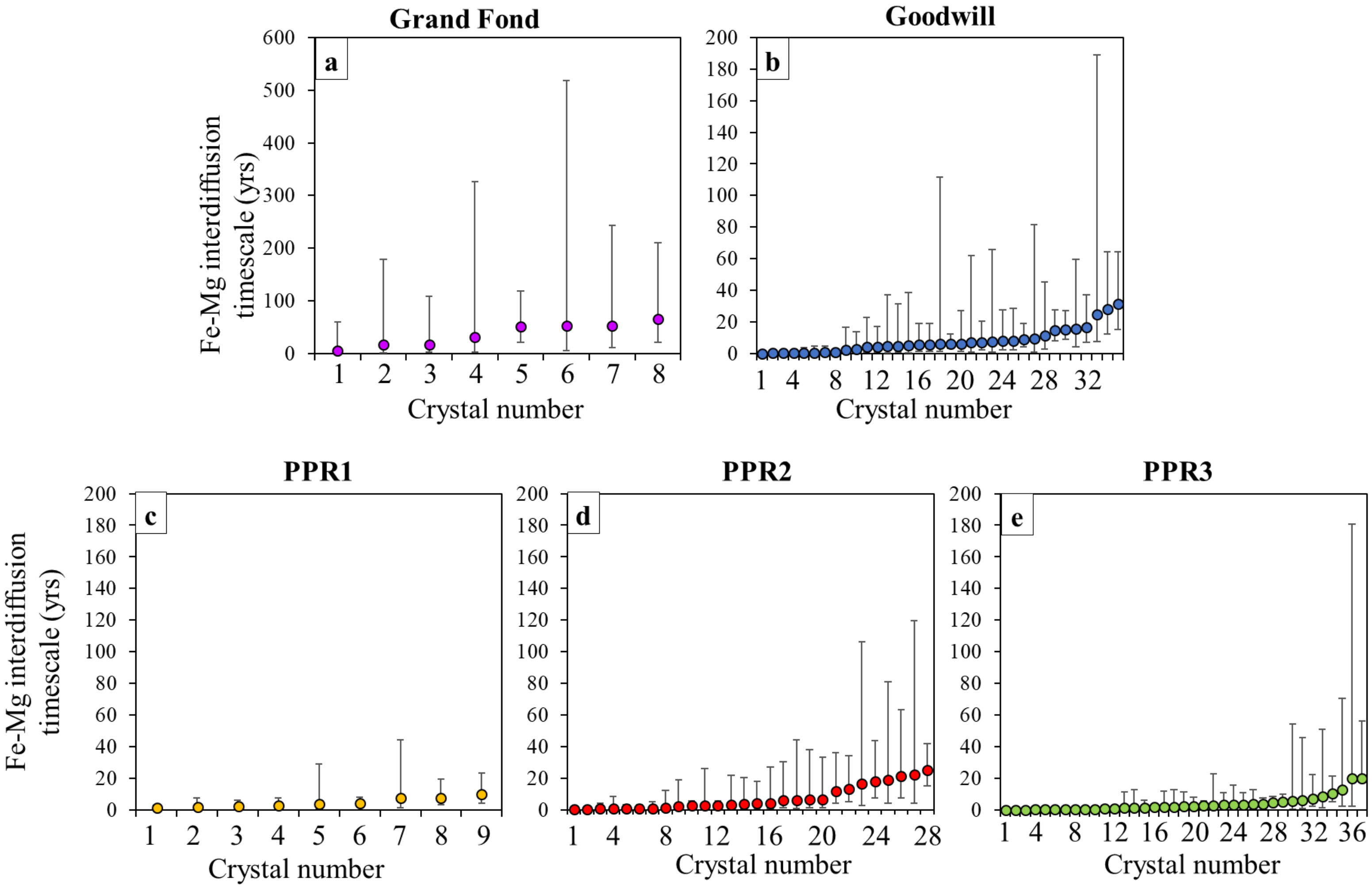

Supplementary Figure S5: Individual timescales and their absolute uncertainties based on the propagation of a temperature uncertainty of ± 25[3] for Grand Fond opx and 9 °C[4] for the small explosive eruptions and interdiffusion coefficient measurements uncertainties on

Grand Fond opx (a), Goodwill (b), PPR1-3 (c-e). These uncertainties are due to the diffusivity calculations (D_0, the interdiffusion coefficient, the activation energy E_a), temperature and resolution of grayscale values of the BSE images[5,6] and are calculated thanks to a Monte Carlo simulation. These uncertainties are asymmetric, with a larger positive error bar and a smaller negative error bar, due to the logarithmic effect of the uncertainties on mainly temperature and diffusivity calculations[7]. The error bars show a high variability, especially for the longest timescales, which places a limit on the statistics of the modelled timescales and their significance.

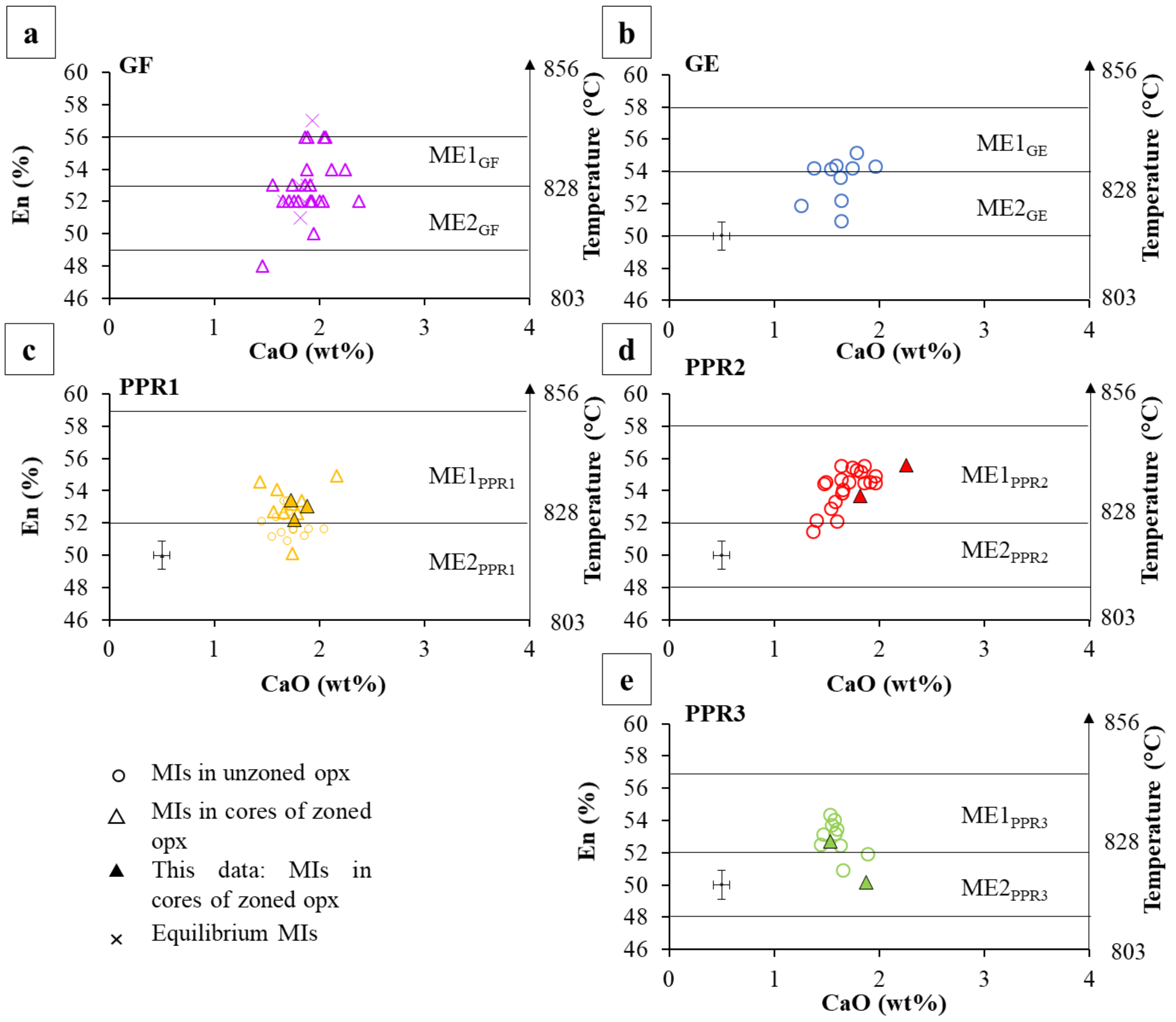

Supplementary Figure S6: En content (%) of the opx hosting the melt inclusions (MI) vs CaO content (wt%) of the MI, represented as a function of the defined magmatic environments (ME) as in Figure 6 (a. Grand Fond (GF); b. Goodwill (GE); c. PPR1; d. PPR2. e. PPR3). The En content/temperature relationship has been added for Grand Fond and extrapolated for the four other eruptions[3]. Filled triangles: this study. Data in empty symbols are MI from another study on the same small explosive eruptions[4]. For Grand Fond, data points with equilibrium MI calculated (crosses)[8].

Supplementary Note: previous results: compositions of natural products and pre-eruptive magma storage conditions

Mineralogy

Samples from the five eruptions (Grand Fond, Goodwill, PPR1, PPR2, PPR3) have been studied in terms of whole-rock compositions, crystal content, melt inclusions (MI) and residual glass compositions in major and trace elements[4,9,10]. Grand Fond and the following small explosive eruptions magmas have whole rock compositions between acid andesites and dacites, with 63 wt% SiO_2 and ~4.5-5 wt% Na_2O+K_2O for Grand Fond[3,9,10] (recalculated with total iron as FeO and on anhydrous basis) and 62-63 wt% SiO_2, ~4.5-5 wt% Na_2O+K_2O for the small explosive eruptions[4] (**Supplementary Figure S7**). All the residual glasses are rhyolitic (75.6-78 wt% SiO_2, 5.6-7 wt% Na_2O+K_2O)[4,9,10] (**Supplementary Figure S7**).

Large explosive eruption: Grand Fond

Grand Fond pumices contain ~30 vol% of phenocrysts: plagioclase (plag, ~21 vol%), orthopyroxene (opx, ~5 vol%) and smaller proportions of clinopyroxene (cpx) and Fe-Ti oxides (mgt) (<1 vol%)[3,9]. The anorthite content of the plag varies from An_{46} to An_{94} with rims of An_{50-55}. Opx are hypersthenes; they span a broad enstatite range, from En_{49} to En_{56} with ~80-85% of unzoned crystals and cpx are diopsides with Wo_{44-45} and En_{37-38} [3]. Fe-Ti oxides (mgt) show compositions with ~75-80 wt% FeO_{tot} and ~10 wt% TiO_2 (Mag_{71-75})[3].

Small explosive eruptions: Goodwill, PPR1 to 3

Pumices from the small explosive eruptions have a similar crystal assemblage as Grand Fond, in decreasing order of abundance: plag >> opx >> cpx >> mgt[4]. For PPR1 and PPR3, amphiboles are present in low proportions. The anorthite content of plag varies from An_{42} to An_{70}, the enstatite content for opx is between En_{46} to En_{59}, and the wollastonite content for cpx varies from Wo_{38} to Wo_{44}. Mgt compositions are between Mag_{69} and Mag_{91}[4].

Major elements composition of melt inclusions and matrix glass

Grand Fond: All MI analyzed in opx and plag exhibit a rhyolitic composition and are located in the calc-alkaline field in the K_2O vs SiO_2 diagram (with Al_2O_3 contents between 11 and 15 wt% and low CaO content, between 1 and 2.5 wt%; **Supplementary Figure S8**)[10]. For opx, their Na_2O+ K_2O content varies from 4.9 to 6.5 wt% and their SiO_2 content from 75.6 to 79 wt%. For plag, they show variations in Na_2O + K_2O of 5.0–5.6 wt% for a SiO_2 range of 76.1 to 78.2 wt%. The residual glasses show a SiO_2 content between 76 and 77 wt% and a Na_2O + K_2O content between 5 and 6.5 wt%[10].

Small explosive eruptions: All MI trapped in opx, cpx, plag and mgt have a rhyolitic composition (71-79 wt% SiO_2, 3.5-7 wt% Na_2O+K_2O, **Supplementary Figure S7**)[4]. In the K_2O vs SiO_2, the MI in all the host minerals are in the medium-K subalkali compositional field

(**Supplementary Figure S8)**. In the Al_2O_3, CaO and FeO versus SiO_2 diagrams, the compositions of the MI show clear differentiation trends where compositions become more evolved from plag then cpx and mgt to opx hosted inclusions[4]. The residual glasses also plot in the domain of the melt inclusion compositions[4]. Some additional electron microprobe profile (EPMA) analysis were performed in this study on the MI in the opx of the 355 µm and 250 µm fractions of the small explosive eruptions (**Supplementary Figure S6-8, Supplementary Table S4**).

Geothermometry and barometry

Grand Fond: The crystallization temperature of Grand Fond's magma has been estimated between 855 and 860°C using titanomagnetites and ilmenites couples; with a fO_2 of NNO + 0.6 to + 0.7[3]. Using the opx-melt geothermometer, a temperature range has been calculated, between 870°C to 890°C[11]. The storage pressure of Grand Fond's magmas, using the CO_2-H_2O ratio content on MI hosted by opx, has been estimated: between 1.6 kbar and 2.7 kbar (corresponding to a depth range of ~7-11 km or 6-10 km considering a crustal density of 2.45 or 2.8 g/cm^3), with one inclusion that records a higher pressure of 7.5 kbar (~27 km depth)[10]. The H_2O content in these inclusions is between 4.5 to 6.3 wt%[10]. Phase equilibria experiments have been carried out on samples from Roseau ignimbrite coming from the same volcano[3]. They give a pressure between 300 and 400 MPa (~12-16 km considering a mean rock density of 2.45 g/cm^3 or between 11-15 km for 2.8 g/cm^3), a temperature of 850±25°C °C, a fO_2 of NNO + 1 and ~7 wt% H_20.

Small explosive eruptions: the equilibrium temperatures between MI and their host opx have been calculated using the opx-liquid thermometer[11] and are of 895 ± 9°C for Goodwill, 893 ± 9°C for PPR1, 893 ± 10°C for PPR2 and 886 ± 6°C for PPR3[4]. The MI pressure of entrapments have also been estimated between 50 and 200 MPa, which correspond to a depth range of 2-8 km or 1.8-7.3 km (considering a mean rock density of 2.45 g/cm^3 or 2.8 g/cm^3, respectively), with a melt H_2O content up to 3.25 wt%[4]. All arguments tend to consider that magmas from the small explosive eruptions were stored at a maximum pressure of 200 MPa, though part of the crystal assemblage necessarily grew at pressures higher than 200 MPa before ascent and storage in shallower reservoirs. These storage conditions give us the parameters necessary for diffusion modelling.

Supplementary Note: major elements composition of melt inclusions from this study

Some additional EPMA analysis were performed in this study on the MI in the opx of the 355-500 µm and 250-355 µm fractions of the small explosive eruptions (**Supplementary Figure S6-8**). They have a SiO_2 content between 72 and 79 wt% and Na_2O+ K_2O content that varies from 3.5 to 6.5 wt%. In the K_2O, Al2O_3, CaO and FeO vs SiO_2 diagrams (**Supplementary Figure S8**), the MI analysed in the opx display a K_2O content between 2 to 3 wt%, 11-14 wt% of Al_2O_3, 1-2.5 wt% in CaO and a FeO content between 2-3.5 wt%, apart from two MI of PPR3 from the 355µm

fraction that have a higher FeO content (4-5 wt%) in the FeO vs SiO_2 diagram. These compositions are within the domain as discussed by the study on the small explosive eruptions MI[4].

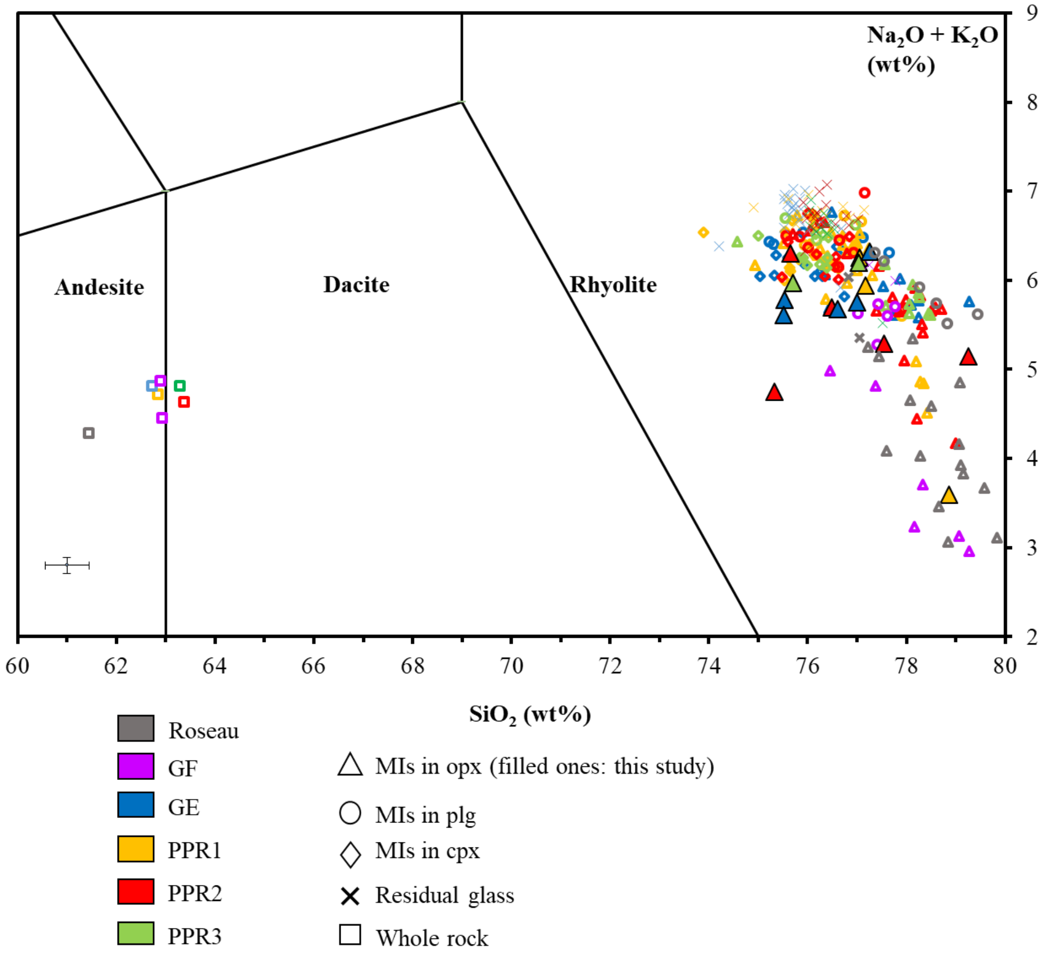

Supplementary Figure S7: Total Alkali Silica (TAS) diagram for MI in opx. Filled triangles: MI in the opx of this study. Empty symbols: analysed MI in opx, cpx, plag in the five eruptions for Grand Fond (GF, purple), Goodwill (GE, blue), PPR1 (orange), PPR2 (red) and PPR3 (green)[4,10]. Crosses: residual glasses of the five eruptions[4,9,10], squares: whole rock compositions of the five eruptions[9]. Data from Roseau ignimbritic eruption is added for comparison[9,10].

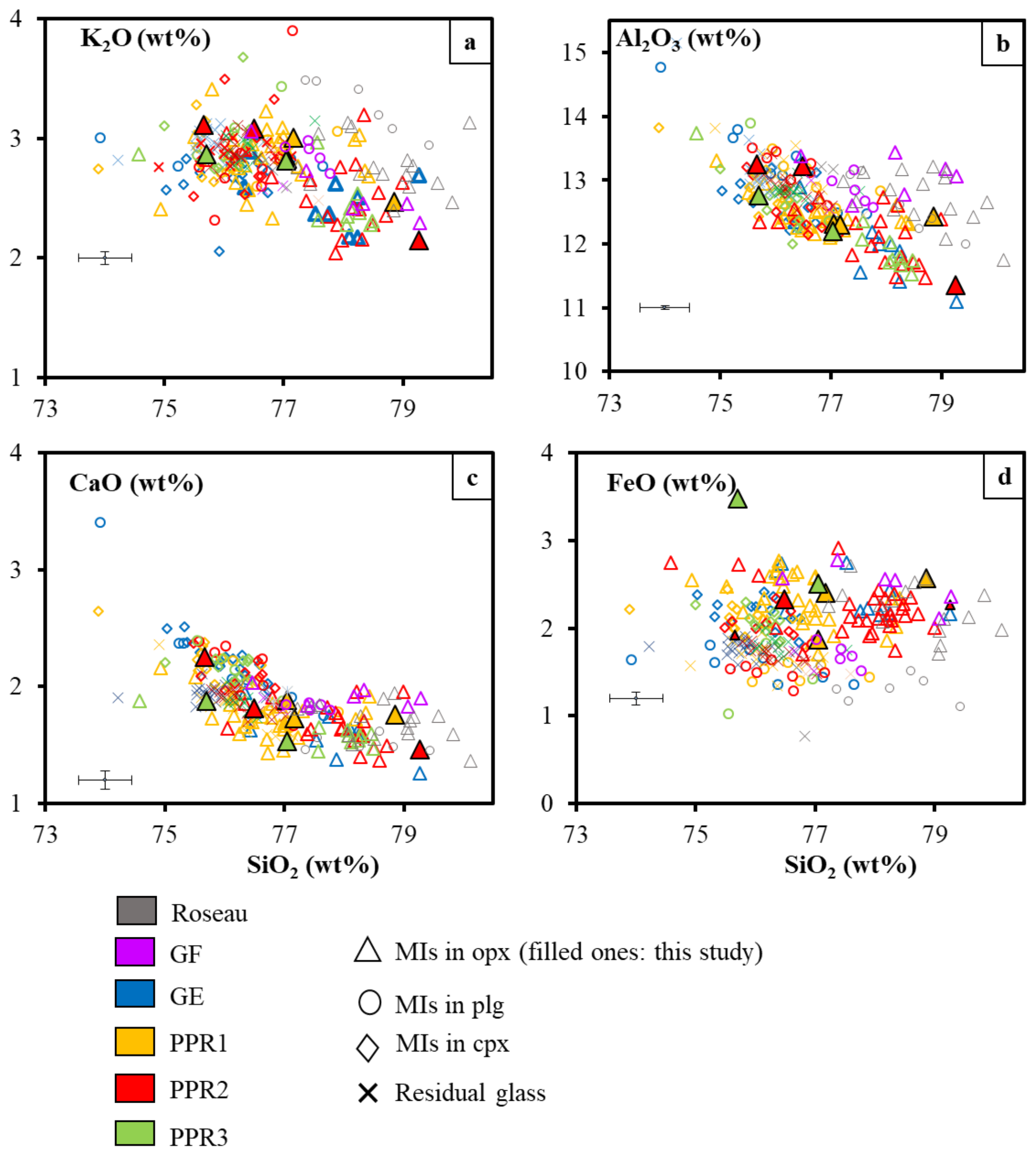
K2O (wt%)
a
Al2O3 (wt%)
b
CaO (wt%)
c
FeO (wt%)
d
SiO2 (wt%)
SiO2 (wt%)
Roseau
GF
GE
PPR1
PPR2
PPR3
MIs in opx (filled ones: this study)
MIs in plg
MIs in cpx
Residual glass

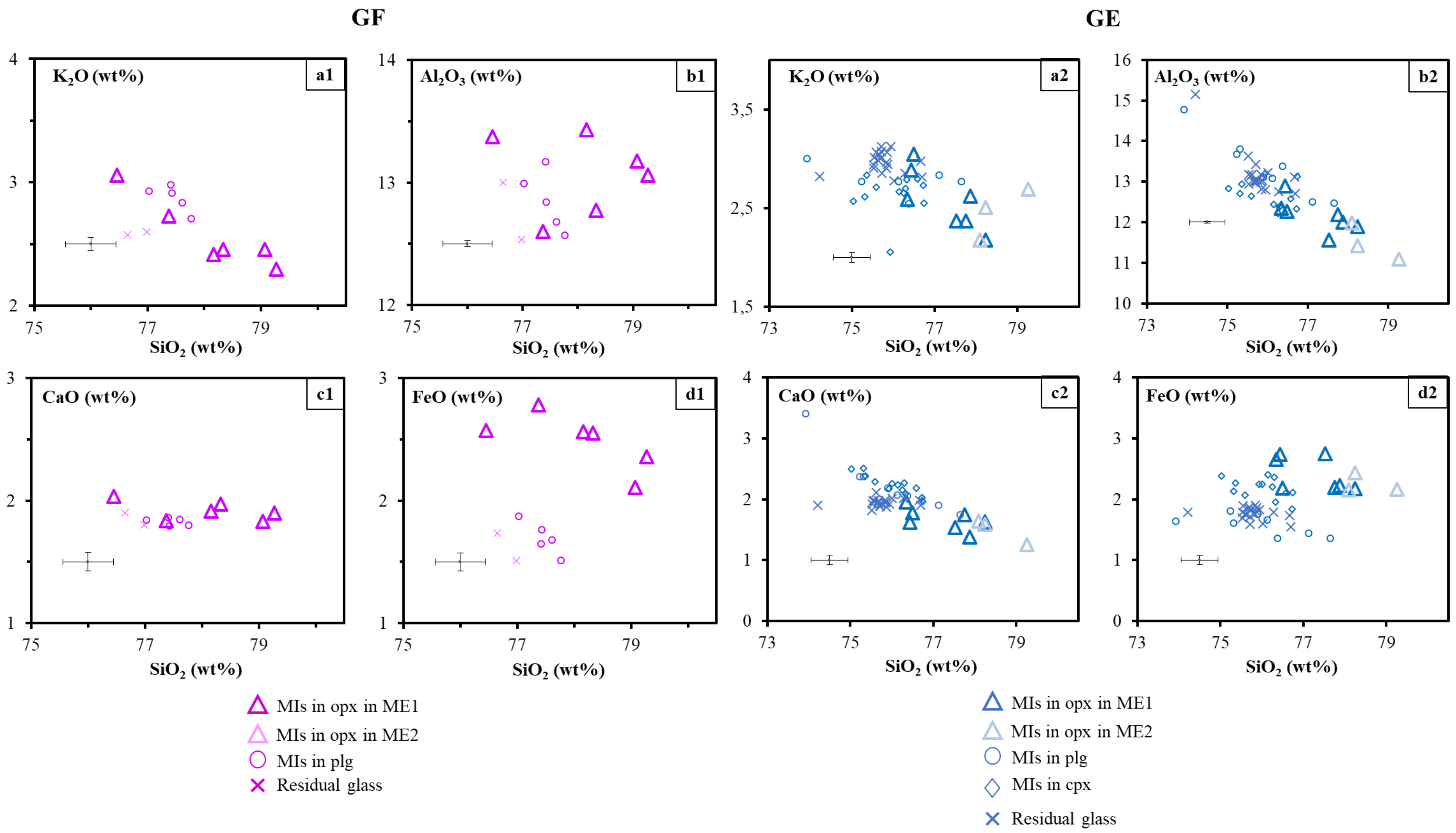
GF
GE
K2O (wt%)
a1
Al2O3 (wt%)
b1
K2O (wt%)
a2
Al2O3 (wt%)
b2
CaO (wt%)
c1
FeO (wt%)
d1
CaO (wt%)
c2
FeO (wt%)
d2
SiO2 (wt%)
MIs in opx in ME1
MIs in opx in ME2
MIs in plg
Residual glass
MIs in opx in ME1
MIs in opx in ME2
MIs in plg
MIs in cpx
Residual glass

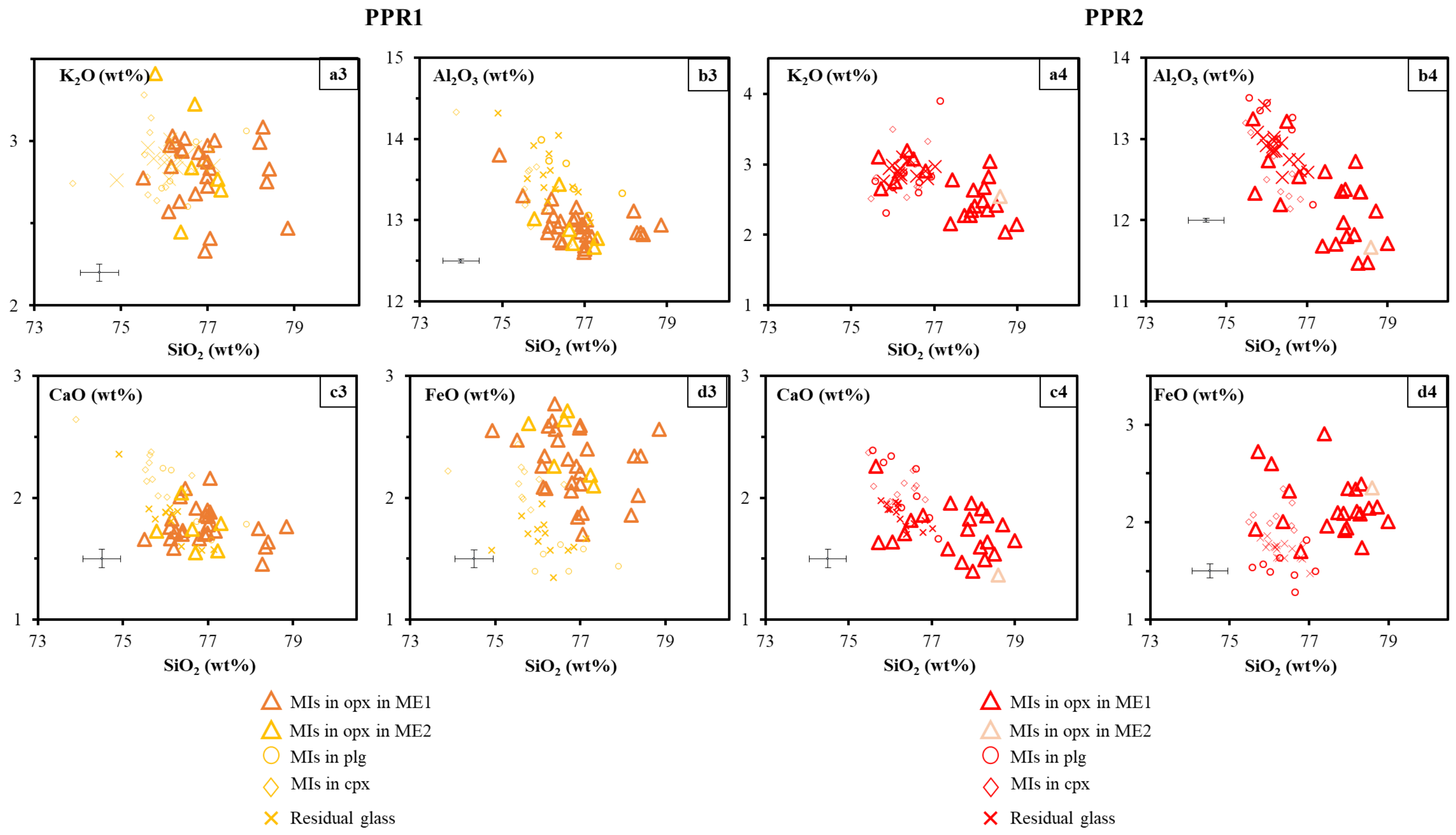
PPR1
PPR2
K2O (wt%)
a3
Al2O3 (wt%)
b3
K2O (wt%)
a4
Al2O3 (wt%)
b4
CaO (wt%)
c3
FeO (wt%)
d3
CaO (wt%)
c4
FeO (wt%)
d4
SiO2 (wt%)
MIs in opx in ME1
MIs in opx in ME2
MIs in plg
MIs in cpx
Residual glass

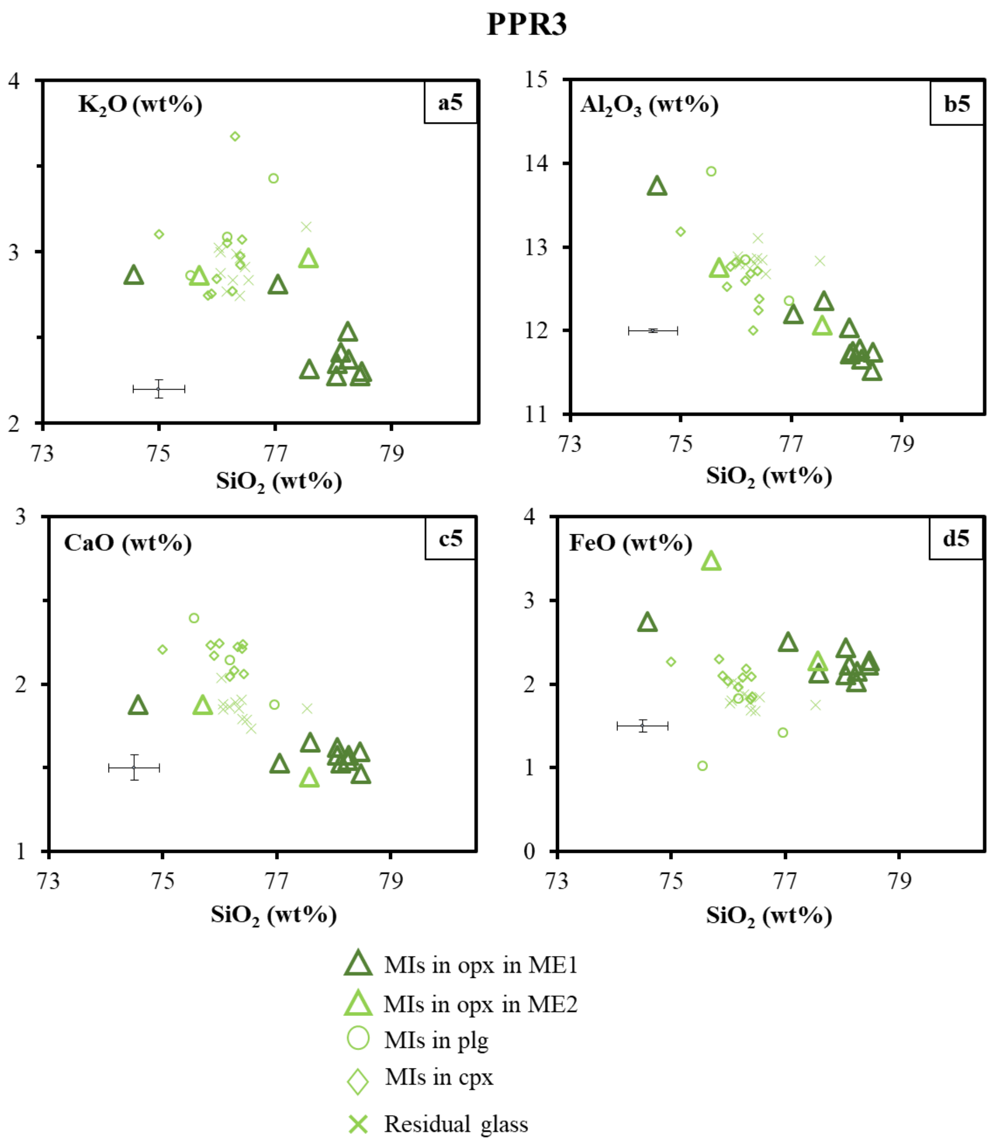

Supplementary Figure S8: Harker diagrams for MI in opx from this study, plag and cpx and residual glasses. a. K_2O vs SiO_2; b. Al_2O_3 vs SiO_2; c. CaO vs SiO_2 and d. FeO vs SiO_2. Roseau (grey), Grand Fond (GF, purple), Goodwill (GE, blue), PPR1 (orange), PPR2 (red) and PPR3 (green). Triangles: analysed MI in opx according to the magmatic environments (ME) defined in **Figure 3** and **4** in which their cores are (either ME1 or ME2). Circles: plag, diamonds: cpx in the five eruptions[4,10] and crosses: residual glasses of the five eruptions[4,9,10]. **a1-d1**: same graphs as before detailed for Grand Fond, **a2-d2**: Goodwill, **a3-d3**: PPR1, **a4-d4**: PPR2 and **a5-d5**: PPR3, with MI in opx.

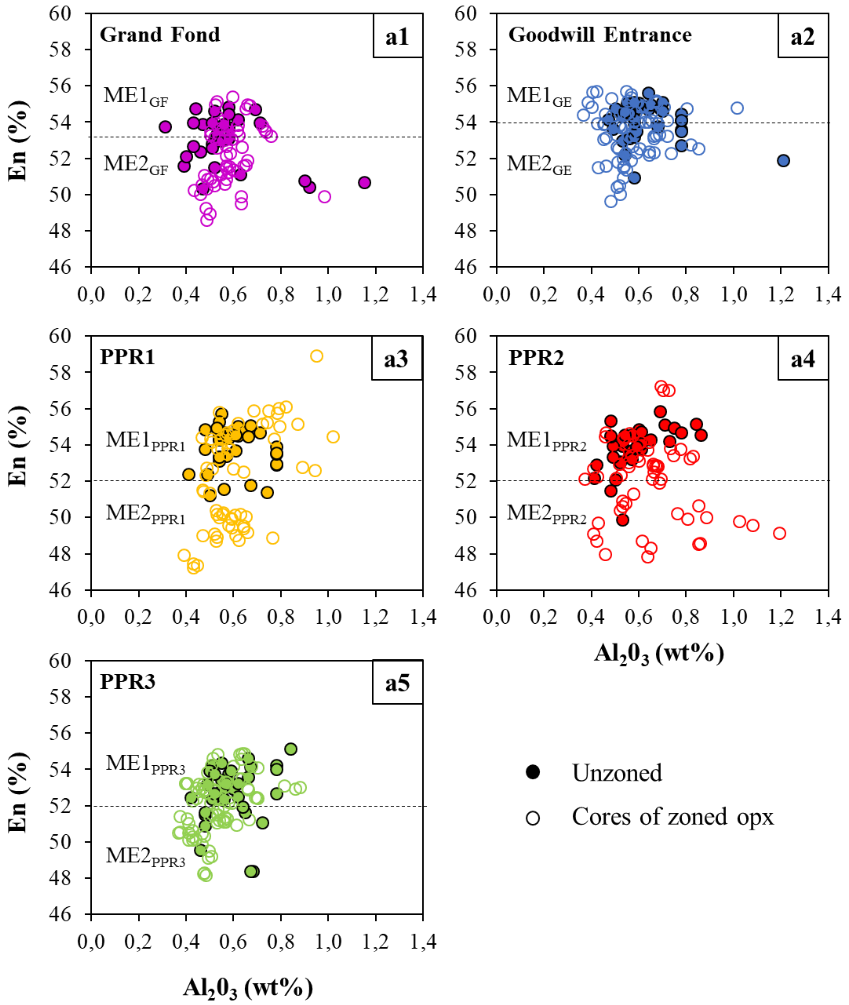

Supplementary Figure S9: Compositions of the unzoned opx compared the cores of the zoned opx in En content (%) vs Al_2O_3 (wt%) from EPMA analyses (a. Grand Fond, b. Goodwill, c. PPR1, d. PPR2, e. PPR3). For all eruptions, unzoned compositions in Al_2O_3 (wt%) are in the

same domain as the majority of the cores of zoned opx (between 0,4-0,8 wt% in Al_2O_3). Their En content also overlap the core compositions of the zoned opx.

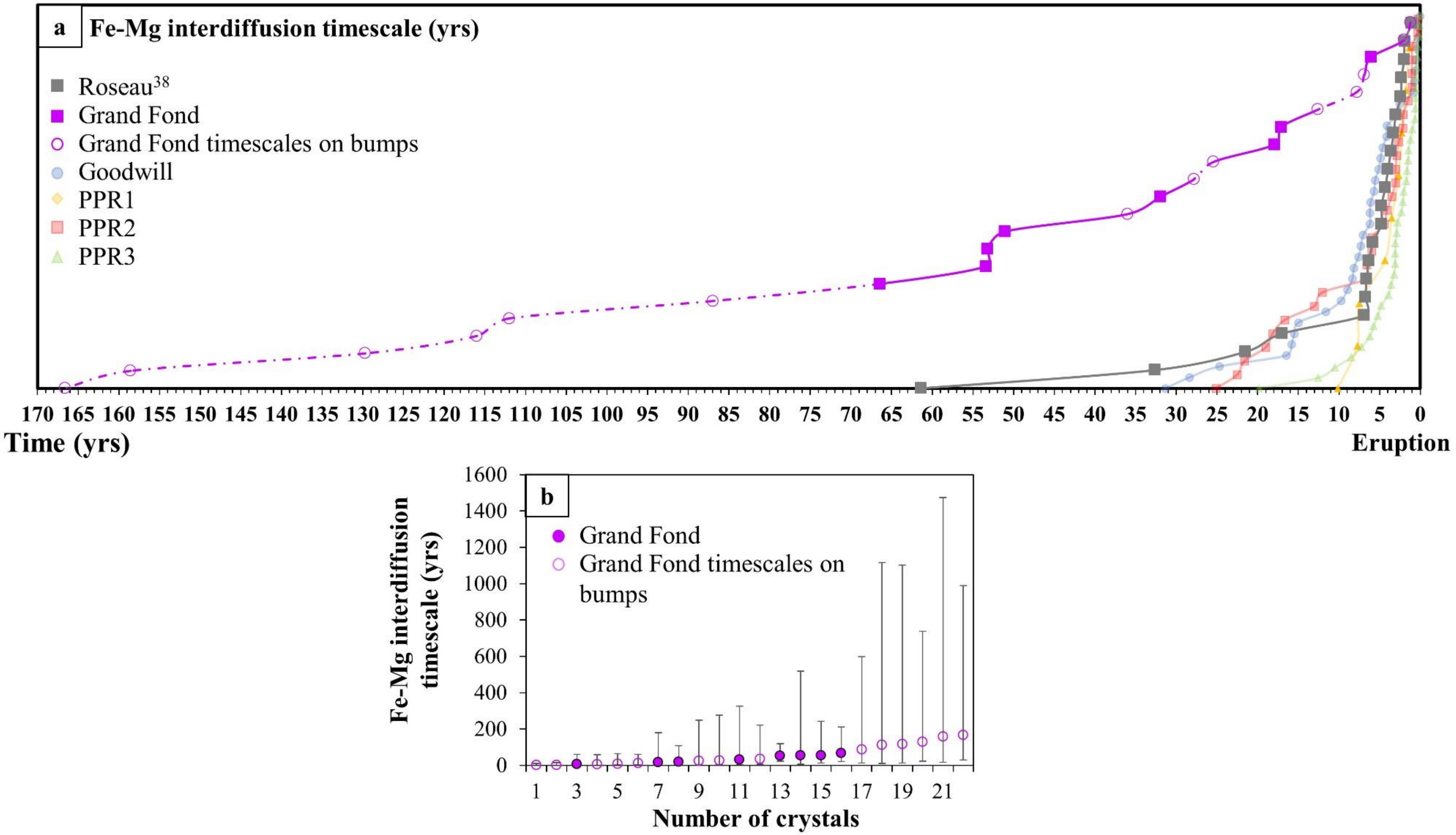

Supplementary Figure S10: a. Rank ordering of the interdiffusion timescales modelled in the opx of Grand Fond, Roseau and the small eruptions, as in Figure 7.f. Timescales modelled on Grand Fond's bumps were added for comparison (**Supplementary Table S3**). **b. Individual**

timescales and their absolute uncertainties based on the propagation of a temperature uncertainty of ± 25 °C[4] and interdiffusion coefficient measurements uncertainties on Grand Fond opx. These uncertainties are due to the diffusivity calculations (D_0, the interdiffusion coefficient, the activation energy E_a), temperature and resolution of grayscale values of the BSE images[5,6] and are calculated thanks to a Monte Carlo simulation. These uncertainties are asymmetric, with a larger positive error bar and a smaller negative error bar, due to the logarithmic effect of the uncertainties on mainly temperature and diffusivity calculations[7]. The error bars show a high variability, especially for the longest timescales, which places a limit on the statistics of the modelled timescales and their significance.

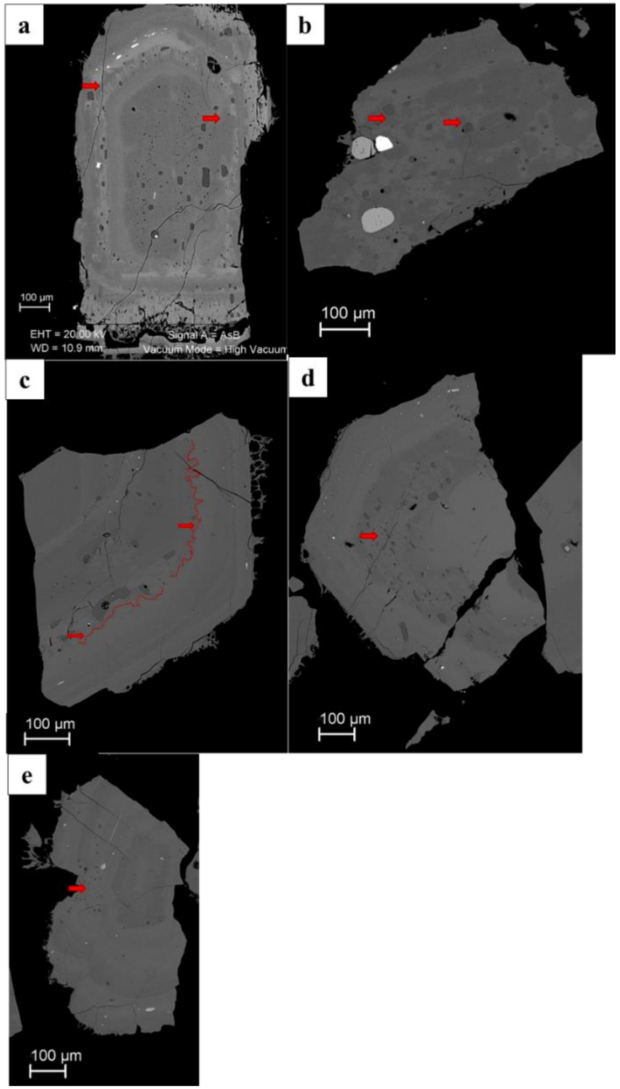

Supplementary Figure S11: Sieve textures in plag crystals of the five eruptions (highlighted by the red arrows; a. Grand Fond, b. Goodwill, c. PPR1, d. PPR2, e. PPR3, red arrows). For plag compositions: see other studies[3,4].

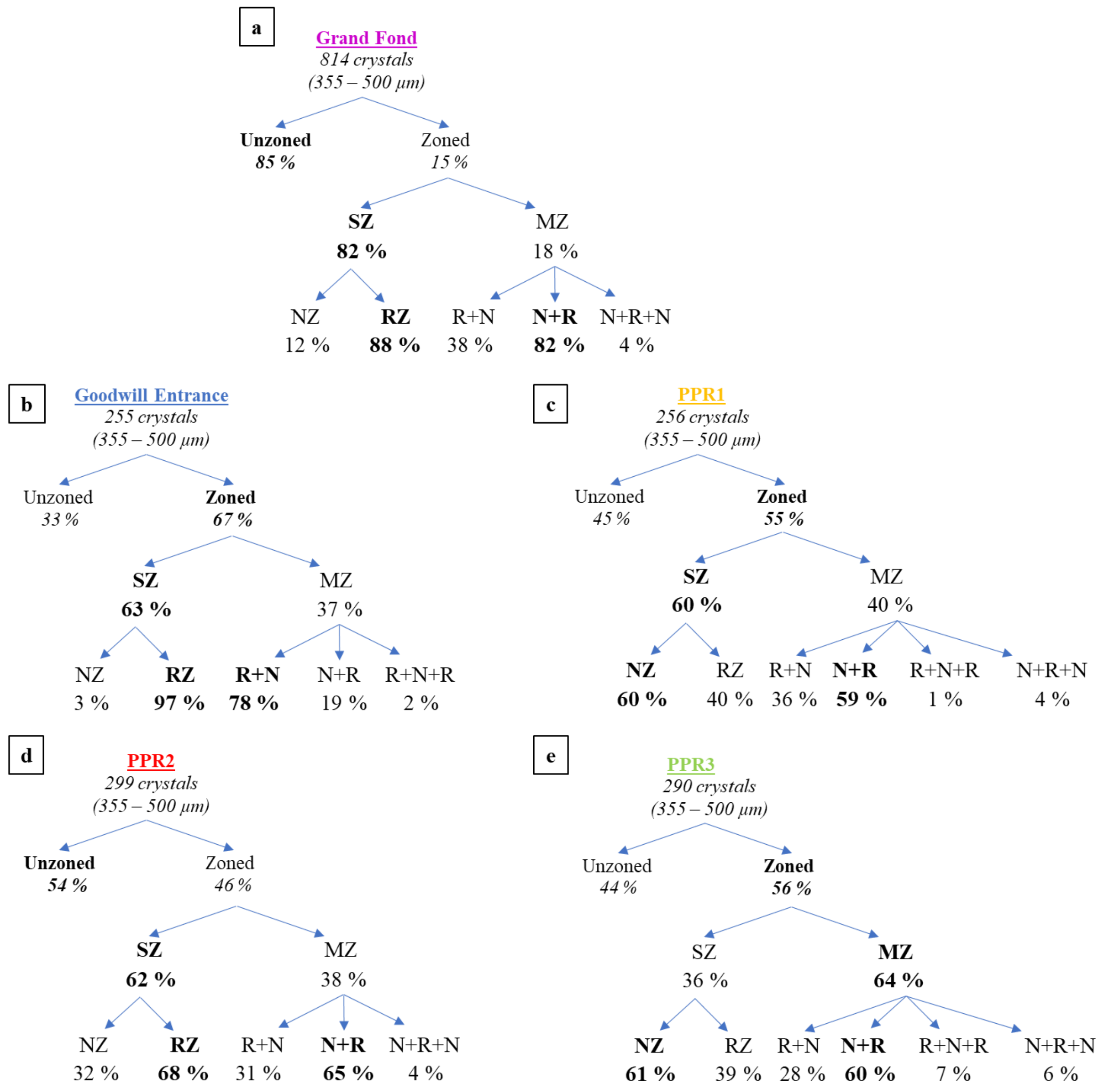

Supplementary Figure S12: Proportions of the zonations identified in the opx of Grand Fond (a) and the small explosive **eruptions (b-e) of**

the 355 µm fraction (same abbreviations as in Figure 2). This Figure has been drawn using Microsoft Office suite 2019 Version 1808 (https://www.microsoft.com/fr-fr/microsoft-365).

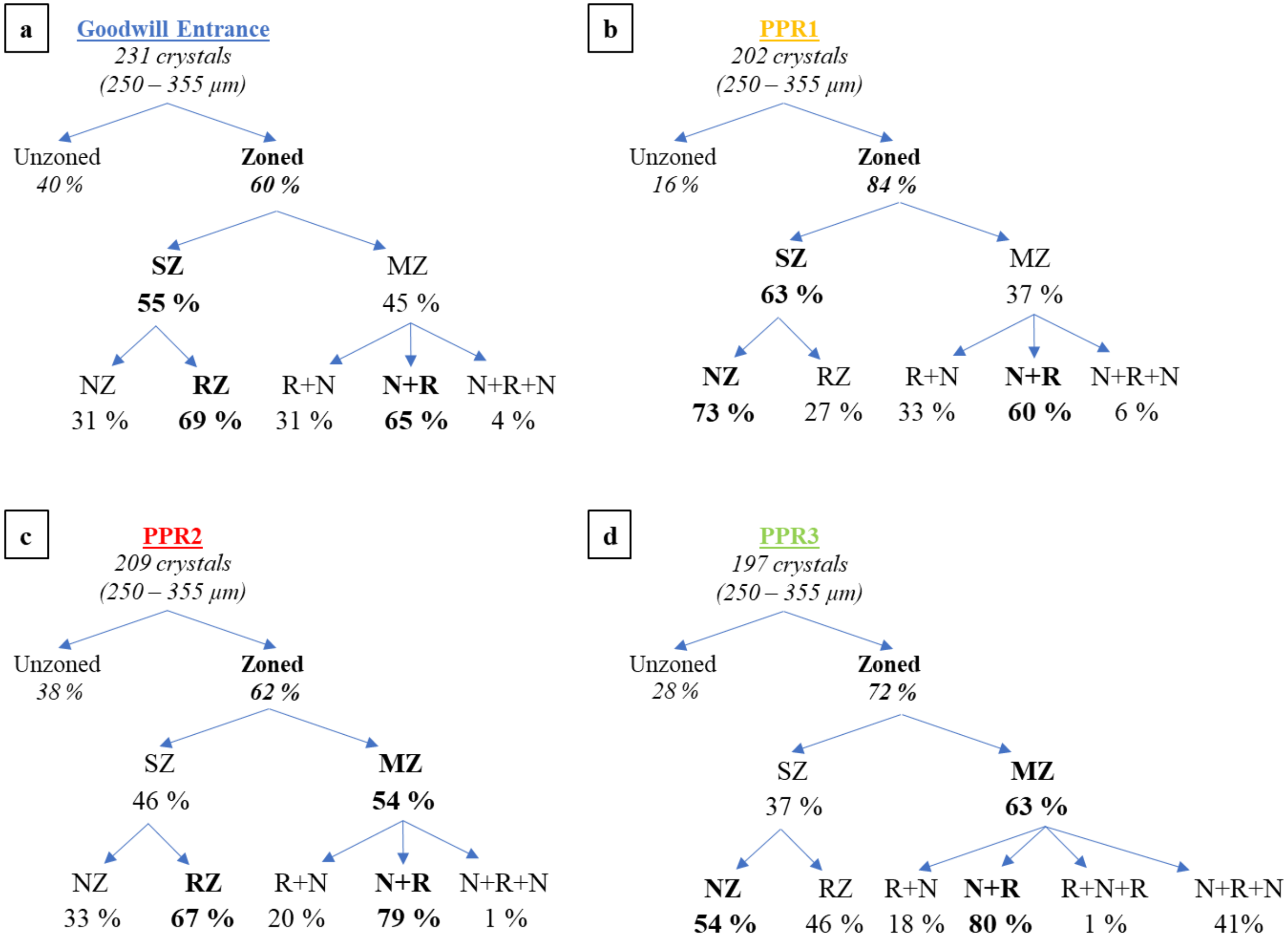

Supplementary Figure S13: Proportions of the zonations identified in the opx of the small explosive**eruptions (a-d) of the 250 µm fraction.**

This Figure has been drawn using Microsoft Office suite 2019 Version 1808 (https://www.microsoft.com/fr-fr/microsoft-365).

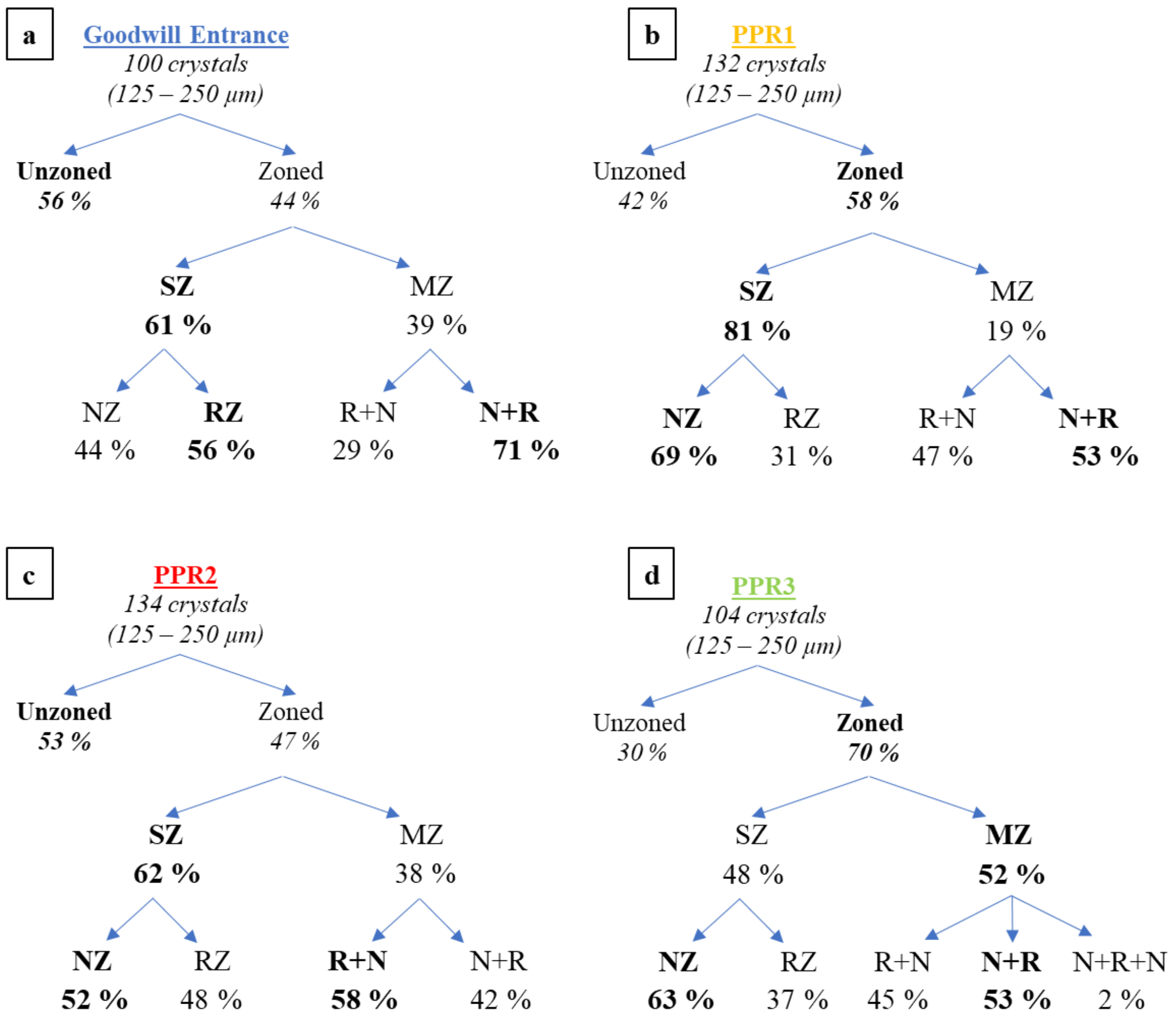

Supplementary Figure S14: Proportions of the zonations identified in the opx of the small explosive **eruptions (a-d) of the 125 µm fraction.**

This Figure has been drawn using Microsoft Office suite 2019 Version 1808 (https://www.microsoft.com/fr-fr/microsoft-365).

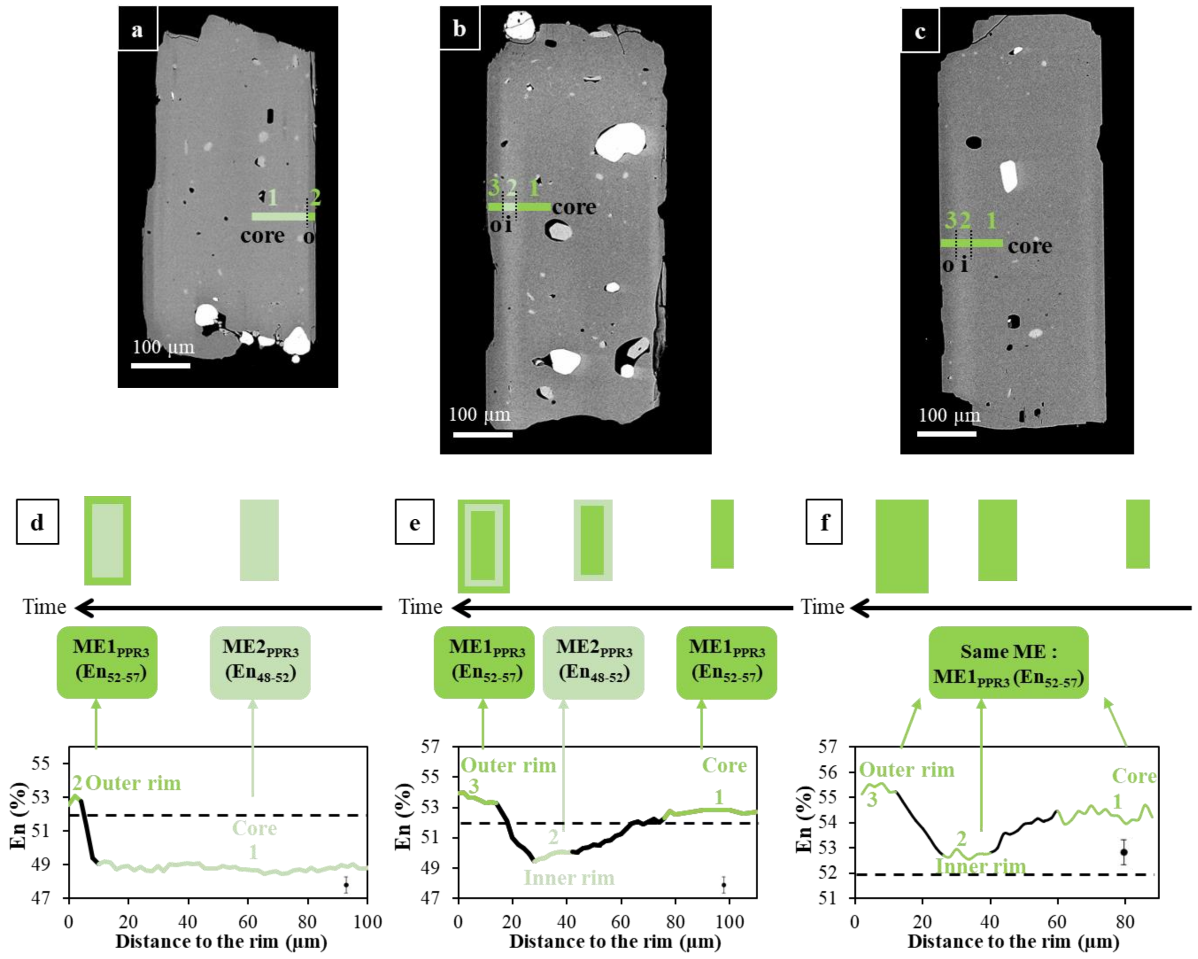

Supplementary Figure S15: Diagram explaining the identification of magmatic environments (ME) from the EPMA compositional profiles after deducing them from the frequency histograms of the En content of all the opx (Figure 3). a, b, c. Back Scattered Electron images of a

zoned opx from PPR3, with EPMA profiles indicated with a green line; cores, inner and outer rims are represented (i: inner rim and o: outer rim). **a.** single-zoned opx; **b-c:** multiple-zoned opx; **d-f:** EPMA profiles acquired in **a-c** in terms of En content (%) with the different plateaus in green that can be linked to different storage conditions corresponding to different ME. **d.** the plateaus (core then outer rim - first plateau) correspond to the two ME identified for PPR3: $ME1_{PPR3}$: En_{52-57} and $ME2_{PPR3}$: En_{48-52}; **e**. the plateaus (core then inner rim - first plateau and outer rim – second plateau) correspond to ME identified for PPR3 and the same as in **d**: $ME1_{PPR3}$ = En_{52-57} and then $ME2_{PPR3}$ = En_{48-52} then back to $ME1_{PPR3}$ = En_{52-57}; **f.** the core and rims have En contents between En_{53-56}, the ME corresponding to these variations is $ME1_{PPR3}$ = En_{52-57}. This zoned opx is then a convecting opx in the same ME. By studying the crystals from the core towards the rim, the history of the crystals and the melt containing them in the reservoir before the eruption can thus be reconstructed. Assuming that the growth of the crystal was from the core to the rims, the first ME known by the crystal would therefore be recorded by the core, as it is shown above the EPMA profile, with the evolution of a crystal in time. This Figure has been drawn using Microsoft Office suite 2019 Version 1808 (https://www.microsoft.com/fr-fr/microsoft-365).

Tables

Supplementary Table S1: Excel file with major element analyses (wt%), En content and Mg number (Mg#) in zoned opx of Grand Fond, Goodwill, PPR1, 2 and 3 (in the three fractions) and all associated profiles in En content vs distance from the rim (µm), as well as major element analyses (wt%) in unzoned opx of Grand Fond, Goodwill, PPR1-3, En content and Mg number (Mg#). Names of the samples: DOM43a1 for Grand Fond, DOM48c2 for Goodwill, DOM51b2 for PPR1, DOM49d2 for PPR2 and DOM49i2 for PPR3.

		Grand Fond	Goodwill	PPR1	PPR2	PPR3
**Value**		**53**	**54**	**52**	**52**	**52**
**Unzoned**		Peak: En_{53-54}	Peak: En_{54-55}	Peak: En_{52-55}	Peak: En_{52-55}	Peak: En_{52-54}
**Population 1**	SZ	$En_{53} \rightarrow En_{55}$	$En_{54} \rightarrow En_{56}$	$En_{54} \rightarrow En_{52}$	$En_{52} \rightarrow En_{55}$	$En_{54} \rightarrow En_{52}$ and En_{57}
	MZ	$En_{53} \rightarrow En_{50} \rightarrow En_{55}$	$En_{54} \rightarrow En_{52} \rightarrow En_{55}$	$En_{52-54} \rightarrow En_{52} \rightarrow En_{54}$	$En_{53-54} \rightarrow En_{52} \rightarrow En_{54}$	$En_{53} \rightarrow En_{51} \rightarrow En_{53}$
**Population 2**	SZ	$En_{51} \rightarrow En_{55}$	$En_{53} \rightarrow En_{56}$	$En_{50} \rightarrow En_{54}$	$En_{50} \rightarrow En_{54}$	$En_{49} \rightarrow En_{54}$
	MZ	$En_{52} \rightarrow En_{54} \rightarrow En_{52}$	$En_{53} \rightarrow En_{54} \rightarrow En_{56}$	$En_{50} \rightarrow En_{48} \rightarrow En_{46}$ or $En_{51} \rightarrow En_{52}$	$En_{49} \rightarrow En_{52} \rightarrow En_{54}$	$En_{51} \rightarrow En_{50} \rightarrow En_{53}$

Supplementary Table S2: En content peaks of the unzoned opx and major En change depending on the two populations of opx for the five eruptions, according to Figure 3 and 4. SZ: single-zoned opx, MZ: multiple-zoned opx.

Supplementary Table S3: Excel file with timescales estimates for Grand Fond (for a temperature of 850°C[3,8]), Goodwill, PPR1-3 eruptions (for a temperature of 890°C[4]) with references to the different pathways and ME changes recorded (name of the samples: DOM43a1 for Grand Fond, DOM48c2 for Goodwill, DOM51b2 for PPR1, DOM49d2 for PPR2 and DOM49i2 for PPR3). The fraction in which the opx has been studied is given (either in the 355-500 µm (355), 250-355 µm (250) or 125-250 µm (125)). Uncertainties associated to the timescales are given (negative error, σ- and positive error, σ+). The type of zonation (reverse or normal) is detailed and the position within the opx (core to rim: single-zoned (SZ) opx or core to inner rim or inner to outer rim for multiple-zoned (MZ) opx). The ME changes are displayed. Grand Fond tmescales modelled on bumps are added in Grand Fond's tab for comparison.

**Eruption**	**Grand Fond**		**Goodwill**		**PPR1**		**PPR2**		**PPR3**	
**ME**	ME1	ME2	ME1	ME2	ME1	ME2	ME1	ME2	ME1	ME2
**Unzoned (%)**	59	26	32	8	33	2	46	3	30	6
**Population 1 SZ (%)**	2		10		25		20		16	
**Population 1 MZ (%)**	2		14		18		16		26	
**Population 2 SZ (%)**		10		26		17		8		9
**Population 2 MZ (%)**		1		11		5		7		13
**Total (%)**	**62**	**38**	**55**	**45**	**76**	**24**	**82**	**18**	**72**	**28**

Supplementary Table S4: Percentages of magma volumes in the two ME for each eruption, estimated by the proportions of unzoned and zoned opx (on the one hand, from the proportions of Figure 2 and on the other hand, from the histograms in Figure 3 and 4: calculated using the number of single-zoned (SZ) and multiple-zoned (MZ) opx of the two populations).

Eruption	Crystal	Fraction	SiO_2	TiO_2	Al_2O_3	FeO	MnO	MgO	CaO	Na_2O	K_2O	P_2O_5	Total
**MI in opx**		(in µm)											
GE	L3C5_R	355	77,01	0,16	12,54	2,38	0,17	0,21	1,73	2,89	2,86	0,06	100
GE	L2C6	355	75,53	0,30	12,87	3,13	0,10	0,22	2,06	2,69	3,09	0,01	100
GE	L6C24_R	355	76,60	0,36	12,22	2,69	0,05	0,25	2,11	3,28	2,39	0,05	100
PPR2	L3C22_R	355	75,32	0,26	11,84	3,83	0,10	1,09	2,34	2,53	2,22	0,48	100
PPR2	L4C7	355	77,54	0,21	12,60	2,38	0,07	0,19	1,67	2,82	2,47	0,05	100
PPR3	L9C1_R	355	77,25	0,15	12,32	2,09	0,11	0,16	1,60	3,23	3,09	0,01	100
PPR3	L9C13_R	355	75,51	0,20	11,81	3,96	0,14	1,04	1,74	3,32	2,29	0,00	100
PPR1	L7C30_c	250	77,05	0,21	12,31	1,87	0,14	0,26	1,88	3,42	2,83	0,02	100
PPR1	L7C30_h2	250	51,74	0,15	0,60	26,85	0,97	18,48	1,09	0,04	0,09	0,00	100
PPR1	L7C29mic	250	78,85	0,26	12,44	2,57	0,13	0,40	1,76	1,12	2,47	0,00	100
PPR1	L7C29_h1	250	51,78	0,19	0,61	27,47	1,21	17,64	1,05	0,01	0,01	0,03	100
PPR1	L7C29mi2c	250	77,16	0,14	12,30	2,40	0,13	0,21	1,73	2,94	3,00	0,00	100
PPR1	L7C29_h2	250	51,98	0,19	0,61	26,86	1,16	18,13	1,03	0,05	0,00	0,00	100
PPR2	L3C22mi_c	250	75,66	0,21	13,25	1,93	0,04	0,30	2,26	3,19	3,11	0,06	100
PPR2	L3C22_h1	250	51,98	0,01	0,76	26,15	0,81	19,26	1,00	0,00	0,01	0,02	100
PPR2	L4C11mi1_c	250	76,49	0,26	13,22	2,32	0,02	0,17	1,82	2,62	3,08	0,00	100
PPR2	L4C11h1_h	250	51,15	0,08	0,48	27,47	0,99	18,75	1,07	0,00	0,00	0,00	100
PPR2	L5C19mi_c	250	79,26	0,23	11,35	2,27	0,07	0,15	1,46	3,00	2,15	0,07	100
PPR3	L9C18mi_c	250	77,04	0,25	12,21	2,51	0,09	0,19	1,53	3,38	2,81	0,00	100
PPR3	L9C18_h	250	51,20	0,10	0,50	27,89	1,09	18,19	0,94	0,04	0,03	0,02	100
PPR3	L13C2mi1_c	250	75,70	0,00	12,76	3,48	0,03	0,21	1,88	3,10	2,87	0,00	100
PPR3	L13C2_h	250	51,26	0,14	0,43	29,00	1,03	17,08	1,01	0,02	0,04	0,00	100

Supplementary Table S5: Major element analyses (wt%) in MI and hosts (opx) of Goodwill (GE), PPR1, 2 and 3 in the 355 and 250 µm fractions (name of the samples: DOM48c2 for Goodwill, DOM51b2 for PPR1, DOM49d2 for PPR2 and DOM49i2 for PPR3).

References

1. Ganguly, J. & Tazzoli, V. Fe2+-Mg interdiffusion in orthopyroxene: retrieval from the data on intracrystalline exchange reaction. *Am. Mineral.* **79**, 930–937 (1994).

2. Dohmen, R., Ter heege, J. H., Becker, H.-W. & Chakraborty, S. Fe-Mg interdiffusion in orthopyroxene. *Am. Mineral.* **101**, 2210–2221 (2016).

3. Solaro, C. *et al.* Petrological and experimental constraints on magma storage for large pumiceous eruptions in Dominica island (Lesser Antilles). *Bull. Volcanol.* **81**, 55 (2019).

4. d'Augustin, T. *et al.* Evidence for an Active, Transcrustal Magma System in the Last 60 ka and Eruptive Degassing Budget (H 2 O, CO 2 , S, F, Cl, Br): The Case of Dominica. *Geochemistry, Geophys. Geosystems* **21**, e2020GC009050 (2020). doi: 10.1029/2020GC009050.

5. Morgan, D. . *et al.* Time scales of crystal residence and magma chamber volume from modelling of diffusion profiles in phenocrysts: Vesuvius 1944. *Earth Planet. Sci. Lett.* **222**, 933–946 (2004).

6. Costa, F. & Morgan, D. *Time Constraints from Chemical Equilibration in Magmatic Crystals*. *Timescales of Magmatic Processes: From Core to Atmosphere* (John Wiley & Sons, Ltd, 2010). doi:10.1002/9781444328509.ch7.

7. Solaro, C. *et al.* A System Dynamics Approach to Understanding the deep Magma Plumbing System Beneath Dominica (Lesser Antilles).

Front. Earth Sci. **8**, 574032 (2020). doi: 10.3389/feart.2020.574032.

8. Solaro-Müller, C. Storage conditions and dynamics of magma reservoirs feeding the major pumiceous eruptions of Dominica (Lesser Antilles Arc). PhD thesis. Paris (France): University Paris Diderot (Sorbonne Paris Cité), 330 (2017).

9. Boudon, G., Balcone-Boissard, H., Solaro, C. & Martel, C. Revised chronostratigraphy of recurrent ignimbritic eruptions in Dominica (Lesser Antilles arc): Implications on the behavior of the magma plumbing system. *J. Volcanol. Geotherm. Res.* **343**, 135–154 (2017).

10. Balcone-Boissard, H. *et al.* Deep pre-eruptive storage of silicic magmas feeding Plinian and dome-forming eruptions of central and northern Dominica (Lesser Antilles) inferred from volatile contents of melt inclusions. *Contrib. to Mineral. Petrol.* **173**, 101 (2018).

11. Putirka, K. D. Thermometers and Barometers for Volcanic Systems. *Rev. Mineral. Geochemistry* **69**, 61–120 (2008).
